# Identification and expression profiling of neuropeptides and neuropeptide receptor genes in a natural enemy, *Coccinella septempunctata*

**DOI:** 10.3389/fphys.2024.1464989

**Published:** 2024-10-09

**Authors:** ShunDa Han, JunJie Chen, ZhaoHan Liu, MaoSen Zhang, PengHui Guo, XiaoXiao Liu, LongRui Wang, ZhongJian Shen, LiSheng Zhang

**Affiliations:** ^1^ College of Horticulture and Landscape Architecture, Tianjin Agricultural University, Tianjin, China; ^2^ State Key Laboratory for Biology of Plant Diseases and Insect Pests, Key Laboratory of Natural Enemy Insects, Ministry of Agriculture and Rural Affairs, Institute of Plant Protection, Chinese Academy of Agricultural Sciences, Beijing, China; ^3^ Key Laboratory of Animal Biosafety Risk Prevention and Control (North) of Ministry of Agriculture and Rural Affairs, Shanghai Veterinary Research Institute, Chinese Academy of Agricultural Sciences, Shanghai, China

**Keywords:** neuropeptides, transcriptome, phylogenetic tree, expression analysis, *Coccinella septempunctata*

## Abstract

**Introduction:**

Neuropeptides and their receptors constitute diverse and abundant signal molecules in insects, primarily synthesized and released primarily from neurosecretory cells within the central nervous system Neuropeptides act as neurohormones and euromodulators, regulating insect behavior, lifecycle, and physiology by binding to receptors on cell surface. As a typical natural predator of agricultural pests, the lady beetle, *Coccinella septempunctata*, has been commercially mass-cultured and widely employed in pest management. Insect diapause is a physiological and ecological adaptative strategy acquired in adverse environments. In biological control programs, knowledge about diapause regulation in natural enemy insects provides important insight for improving long-term storage, transportation, and field adoption of these biological control agents. However, little is known about the function of neuropeptides and their receptors in controlling reproductive diapause of *C. septempunctata*. It is unclear which neuropeptides affect diapause of *C. septempunctata*.

**Methods:**

In this study, RNA-seq technology and bioinformatics were utilized to investigate genes encoding neuropeptides and their receptors in female adults of *C. septempunctata*. Quantitative real-time PCR (qRT-PCR) analysis was employed to examine gene expression across different development/diapause stages.

**Results:**

A total of 17 neuropeptide precursor genes and 9 neuropeptide receptor genes were identified, implicated in regulating various behaviors such as feeding, reproduction, and diapause. Prediction of partial mature neuropeptides from precursor sequences was also performed using available information about these peptides from other species, conserved domains and motifs. During diapause induction, the mRNA abundance of *AKH* was notably higher on the 10th day compared to non-diapause females, but decreased by the 20th day. In contrast, *GPHA* showed lower expression levels on the 5th day of diapause induction compared to non-diapause females, but increased significantly by the 15th and 20th days. *NPF* was higher expressed in head and midgut while *DH* showed higher expression in the fat body and midgut. Additionally, *NPF* expression remained consistently lower throughout all stages of diapause induction compared to non-diapause conditions in females.

**Discussion:**

This study represents the first sequencing, identification, and expression analysis of neuropeptides and neuropeptide receptor genes in *C. septempunctata*. Our results could provide a foundational framework for further investigations into the presence, functions, and potential targets of neuropeptides and their receptors, particularly in devising novel strategies for diapause regulation in *C. septempunctata*.

## 1 Introduction

Neuropeptides are pivotal in regulating essential physiological processes such as development, reproduction, circadian rhythm, and feeding in insects. They are predominantly synthesized and released from neurosecretory cells within the central nervous system (CNS) (Nässel and Winther, 2010; Schoofs et al., 2017; Abou El Asrar et al., 2020; Toprak, 2020). Most of the known neuropeptides are oligopeptides or small protein molecules composed of several to tens of amino acids (Schoofs et al., 2017; Abou El Asrar et al., 2020). Biologically active neuropeptides undergo intricate post-translational processing from their precursor forms. Acting as neuromodulators or neurohormones, neuropeptides transmit signals by binding to specific membrane receptors. Most neuropeptide receptors belong to the G protein-coupled receptor family, initiating intracellular cascade reactions upon coupling with G proteins in the cell membrane, ultimately eliciting cellular responses (Audsley and Down, 2015; Rosenbaum et al., 2009). Some neuropeptide receptors are not GPCRs, for example, insulin-like receptor is tyrosine kinase receptors (TKR) (Choi and Bai, 2023). Recently, high-throughput sequencing techniques (transcriptome and genome analysis) and bioinformatics technology has greatly promoted the prediction and identification of insect neuropeptides and their receptors (Tanaka et al., 2014; Wang et al., 2018; Li FF. et al., 2020). Over the past 2 decades, substantial progress has been made in identifying novel neuropeptides and homologous receptors across various insect species of significance in human health, agriculture, and ecological environments. Examples include the discovery of 35 neuropeptides and peptide hormones in *Anopheles gambiae* (Riehle et al., 2002), 37 neuropeptides in *Bombyx mori* (Roller et al., 2008), 36 neuropeptides in *Apis mellifera* (Hummon et al., 2006), and 41 genes encoding neuropeptides and protein hormones in *Tribolium castaneum* (Li et al., 2008).

The lady beetle, *C. septempunctata*, is a natural predator of agricultural pests, and it is economically important for controlling aphids, whiteflies, mites, thrips, and lepidopteran pests (Chen et al., 2023). This beneficial insect has been extensively mass-cultured and deployed in greenhouses and farmlands across Europe, Asia, and North America (Yang et al., 2014). In Northern China, *C. septempunctata* adults enter winter diapause in response to short photoperiods and low temperatures, and our previous work identified the specific conditions that elicit diapause (Wang et al., 2013). Diapause creates challenges and opportunities for biological control. While diapause may interrupt rearing activities and reduce field effectiveness (as diapausing beetles do not feed on pests), regulating diapause can enhance the long-term storage and timely deployment of *C. septempunctata*. Mastering the mechanism of insect diapause and regulating the diapause of natural enemy insects can promote the shelf-life and effective release of mass-produced predatory ladybeetles. Given the pivotal role of neuropeptide signaling systems in physiological behavioral processes, as well as the high specificity and low target interference of neuropeptide receptors, insect neuropeptides are considered ideal targets for cultivating specific insect regulators. Thus, elucidating the structure and function of neuropeptides and their receptors involved in *C. septempunctata* diapause is crucial.

In this study, we employed transcriptome sequencing and bioinformatics analysis to identify genes encoding neuropeptide precursors and their receptors in *C. septempunctata*. Additionally, we conducted structural analysis and predicted the mature peptides of the identified neuropeptide precursors. Neuropeptides were classified into subclasses through phylogenetic analysis. Using qRT-PCR, we examined the expression profiles of partial neuropeptides precursor in five different tissues (head, thorax, fat body, ovary and midgut) of female adults at diapause-inducing/non-diapause inducing condition. The sequences of these identified genes serve as a foundational resource for further exploring the roles of neuropeptides precursor in diapause regulation in *C. septempunctata*.

## 2 Materials and methods

### 2.1 Insect rearing and tissue collection

The *C. septempunctata* laboratory colony initially originated from field-collected adults from the wheat field at the east gate of the Chinese academy of agricultural sciences located in Haidian district, Beijing, China, in 2010. Offspring of those lady beetles were maintained at 24°C ± 1°C and 70% relative humidity (RH) under a long-day photoperiod condition (normal developmental conditions) (16 h:8 h, light/dark) and fed with fresh pea aphids (*Aphis glycines*) daily (Chen et al., 2023; Wang et al., 2013; Li et al., 2022). To induce diapause, newly emerged adults were transferred to an environment with the following conditions: 18°C ± 1°C; 10 L: 14 D (10 h light: 14 h dark per 24 h); RH 70% ± 10% (diapause-inducing conditions).

To evaluate the relative mRNAs expression of the neuropeptides precursor genes and neuropeptide receptor genes at different stages and tissues, we sampled the head, thorax, fat body, ovary and midgut of the newly emerged adults (NE), five-day-old female adults (N5), ten-day-old female adults (N10), fifteen-day-old female adults (N15) and twenty-day-old female adults (N20) of *C. septempunctata* under non-diapausing conditions. We also sampled the head, thorax, fat body, ovary and midgut of five-day-old female adults (D5), ten-day-old female adults (D10), fifteen-day-old female adults (D15) and twenty-day-old female adults (D20) of *C. septempunctata* under diapause-inducing conditions. All samples were collected, cleaned, and frozen using liquid nitrogen. The samples were then stored in a refrigerator (−80 °C) until analyses were done. Each treatment was performed with three biological replicates, and each replicate was made up of six female adults.

### 2.2 RNA extraction and RNA-seq

Total RNA was extracted from each sample using Trizol Reagent (Invitrogen, Waltham, MA, United States), according to the manufacturer’s protocol for insect tissues. The quality and quantity of total RNA in each sample were assessed using an Agilent 2,100 Bioanalyzer (Agilent Technologies, Palo Alto, CA, United States) and NanoDrop (Thermo Fisher Scientific, Waltham, MA, United States). RNA-seq libraries were prepared from the heads of *C. septempunctata*, with four replicate libraries prepared. The libraries were sequenced separately using the DNBSEQ-T7 sequencer.

### 2.3 Transcriptome data analysis

Unigene expression levels were calculated and normalized to RPKM (Reads Per kb per Million reads). Unigene sequences were aligned by BLASTx and TBLASTx searches against the protein database (http://blast.ncbi.nlm.nih.gov/) such as NCBI non-redundant protein (Nr) database, SwissProt database, KEGG Ontholog database (KO) and Gene Ontology (GO) for annotation information. Transcriptomic (RNA-seq) data obtained from *C*. *septempunctata* were utilized to identify neuropeptides precursor and their receptors.

### 2.4 Sequence analysis and phylogenetic tree analysis

Amino acid sequences of neuropeptide sequences of *C. septempunctata* were determined using the ExPASy Translate tool (https://web.expasy.org/translate/). Conserved domain analysis was conducted with the CDD Search program of the National Center for Biotechnology Information (NCBI) web server. To investigate the evolutionary relationships of neuropeptide sequences and neuropeptide receptor, the sequences of various species (Supplementary Tables S1 and S2) were downloaded from the NCBI and aligned with sequences of neuropeptide sequences using ClustalW 2 and ESPript 3.0 webserver. For determining protein sequence similarities and secondary structure information, the model of the neuropeptide protein was constructed with SWISS-MODEL (https://swissmodel.expasy.org/). Phylogenetic trees were constructed using the best-fit nucleotide substitution model (WAG) and a bootstrap analysis with 1,000 replicates in the MEGA v.11 software.

### 2.5 Times and tissues expression analysis of *C. septempunctata* female adults

Total RNA was extracted from female tissues (including head, thorax, fat body ovary and midgut) of *C. septempunctata* under different inducing condition using a RNAisoPlus Total RNA IsolationKit (Takara, Dalian, China) according to the manufacturer’s protocol. The integrity and purity of total RNA in all samples were assessed by 1% agarose gel electrophoresis, and RNA quantity was measured using a P-class Nanophotometer (Implen, Germany). For cDNA synthesis, 1 μg of total RNA was reverse transcribed to cDNA using TransScript^®^One-Step gDNA Removal and cDNA Synthesis SuperMix (TransGen Biotech, Beijing, China) under the following conditions: 42°C for 30 min, and 85°C for 5 s. Subsequently, real-time qRT-PCR was done using TOROGreen^®^ 5G qPCR Premix (Toroid Technology Limited) and a LightCycler^®^ 96 Instrument (Roche, Switzerland). All reactions were run in triplicates, with a total volume of 20 μL, and each reaction consisted of 10 μL TOROGreen Premix, 0.8 μL of each specific primer (Supplementary Table S1), 1 μL sample cDNA, and 7.4 μL nuclease-free water. PCR amplification of the genes was conducted under the following conditions: 95°C for 5 min, followed by 40 cycles at 98°C for 10 s and 55◦C–59°C for 20 s. A dissociation step cycle (95°C for 10 s, 65°C for 60 s, and from 65°C to 97°C at increments of 0.2°C/s; 5 readings/°C) was added for the melting curve analysis. When the reactions were complete, CT values were determined using fixed threshold settings. The relative expression levels of genes were analyzed using the comparative 2^−ΔΔCt^ quantitation method (Livak and Schmittgen, 2001), and abundance was normalized to the *CsActin* transcript level (Li et al., 2008; Li et al., 2022). A total of 3 independent biological samples were included for each group, and three technical replicates of each biological sample were processed for all reactions.

### 2.6 Statistical analysis

Data (mean ± standard error) of the relative expression levels from various samples were subjected to ANOVA (one-way analysis of variance), followed by Tukey’s significance difference tests implemented in SPSS Statistics 17.0 (IBM SPSS Statistics, Chicago, IL, United States). Data (mean ± standard error) of the relative expression levels from various samples were subjected to *t*-test in Graph Pad Prism (Version 9.0). Expression patterns of neuropeptides precursor and neuropeptide receptors across different tissues of female *C. septempunctata* were all visualized using Graph Pad Prism (Version 9.0).

## 3 Results

### 3.1 Transcriptome sequencing and sequence assembly

The total transcriptome sequencing provided approximately 193.5 million reads (19.35 Gb). After removing low-quality, adaptor, and contaminated sequence reads, the clean reads of each sample reach to 6.44 Gb. For annotations of unigenes to Nr, KEGG and GO databases, 14,094 (65.23%) were annotated using the NCBI-Nr database; 13,245 (61.30%) were annotated by KEGG; 7,079 (32.76%) were annotated by GO (Table 1). The raw sequence reads were deposited in NCBI Sequence Read Archive under the accession number PRJNA1154946.

**TABLE 1:** Summary of *C. septempunctata* transcriptome assembly.

Statistics project	Number
Clean reads from all samples	38.7 Gb
Q20 (%)	97.69–97.96
Q30 (%)	92.77–93.59
Nr ration	65.23%
KEGG ration	61.30%
GO ration	32.76%
Total Raw Reads (M)	45.57
Total Clean Reads (M)	42.93–43.16
Total Mapping Ratio (%)	94.19–94.70

### 3.2 Identification of *C. septempunctata* neuropeptides precursor and their receptors

Using the reported neuropeptides precursor and their receptors of other insect species in NCBI as queries BLAST searching was performed. We identified a total of 17 neuropeptide precursor transcripts (Table 2) and 12 receptor transcripts encoding 9 neuropeptide receptors (Table 3) from in the transcriptome databases of *C. septempunctata* head, including *Bursicon* (*Bur*), *Neuropeptide F* (*NPF*), *Short neuropeptide F* (*sNPF*), *FMRFamide neuropeptides* (*FMRF*), *PBAN-type neuropeptides-like* (*PBAN*), *Tachykinin* (*TK*), *Prothoracicostatic peptide* (*PTTH*), *Adipokin hormo* (*AKH*), *Cardioactive peptide* (*CCAP*), *Prohormone-2* (*PH*), *SIFamide neuropeptide* (*SIFamide*), *Diuretic hormone 37* (DH), *Ion transport peptide* (*ITP*), *Glycoprotein hormone alpha 2* (*GPHA*), *Glycoprotein hormone beta-5* (*GPHB*), *Neuropeptide-like precursor* (*NPL*) and *Allatotropin I preprohormone precursor* (AT), *Insulin-like peptide receptor* (*InR*), *Neuropeptides capa receptor* (*CAPAR*), *neuropeptide SIFamide receptor* (*SIFR*), *D. hormone receptor* (*DHR*), *CCHamide-1 receptor* (*CCH1R*), *CCHamide-2 receptor* (*CCH2R*), *Trissin receptor* (*TrR*), *RYamide receptor 1* (RY1R), *RYamide receptor 2* (RY2R), *Sex peptide receptor 1* (SPR) and *Tachykinin-like peptides receptor* (*TKR*).

**TABLE 2:** Neuropeptides precursor identified from *C. septempunctata*.

Gene name	Unigene ID	ORF	Homology search with known protein			
		(aa)	Species	E-value	Accesion No.	Identity (%)
*Bursicon*	Csep033675.1	169	Sitophilus oryzae	2e-87	XP_030754853.1	82.47
*Neuropeptide F*	Csep029464.1	123	*Zophobas atratus*	1e-25	UXO98088.1	53.57
*Short neuropeptide F*	Csep029051	97	*Coccinella septempunctata*	6e-150	XM_044905695.1	100.00
*FMRFamide neuropeptides*	Csep040955	207	*Harmonia axyridis*	4e-111	XP_045478717.1	78.00
*PBAN-type neuropeptides-like*	Csep018229	145	*Nicrophorus vespilloides*	1e-04	XP_017786058.1	96.00
*Tachykinin*	Csep038274	285	*Harmonia axyridis*	6e-115	XP_045478364.1	67.29
*Prothoracicostatic peptide*	Csep039735	191	*Coccinella septempunctata*	0.0	XM_044892713.1	100.00
*Adipokin hormo*	Csep015456	71	*Asbolus verrucosus*	6e-20	RZB41086.1	51.40
*Cardioactive peptide*	Csep001786	145	*Coccinella septempunctata*	3e-70	XP_045465746.1	100
*Prohormone-2*	Csep034584	299	*Tribolium castaneum*	6e-64	XP_044253031.1	44.40
*SIFamide neuropeptide*	Csep012885	71	*Tribolium castaneum*	1e-44	XP_001814498.1	67.69
*Diuretic hormone 37 like protein*	Csep030921	137	*Asbolus verrucosus*	5e-19	RZC31784.1	35.97
*Ion transport peptide*	Csep013401	124	*Coccinella septempunctata*	7e-87	XP_044746445.1	100
*glycoprotein hormone alpha 2*	Csep021738	122	*Tribolium castaneum*	5e-64	NP_001164244.1	73.77
*glycoprotein hormone beta-5*	Csep024296	154	*Asbolus verrucosus*	8e-74	RZB89874.1	85.83
*Neuropeptide-like precursor*	Csep035491	306	*Harmonia axyridis*	8e-132	XP_045477560.1	76.40
*Allatotropin I preprohormone precursor*	Csep007712.1	99	*Tribolium castaneum*	6e-13	XP_008197028.1	41.51

Open Reading Frame.

**TABLE 3:** Neuropeptide receptors identified from *C. septempunctata*.

Gene name	Unigene ID	ORF	Homology search with known protein			
		(aa)	Species	E-value	Accesion No.	Identity (%)
*Insulin-like receptor isoform X1*	Csep018270	1,437	*Coccinella septempunctata*	0.0	XP_044766311.1	100
*Insulin-like peptide receptor 2*	Csep040401	1,107	*Coccinella septempunctata*	0.0	XP_044749880.1	100
*Neuropeptides capa receptor*	Csep006452	644	*Coccinella septempunctata*	0.0	XP_044764663.1	100
*neuropeptide SIFamide receptor*	Csep000244	274	*Coccinella septempunctata*	0.0	XP_044757383.1	97.67
*Diuretic hormone receptor*	Csep024929.1	440	*Harmonia axyridis*	0.0	XP_045474415.1	81.35
*CCHamide-1 receptor*	Csep037149	420	*Coccinella septempunctata*	0.0	XP_044759703.1	100
*CCHamide-2 receptor*	Csep035103	418	*Coccinella septempunctata*	0.0	XP_044758875.1	100
*Trissin receptor2*	Csep032989.1	169	*Harmonia axyridis*	0.0	XP_044750262.1	100
*RYamide receptor 1*	Csep002267.1	193	*Harmonia axyridis*	2e-107	XP_045468643.1	81.96
*RYamide receptor 2*	Csep002854.1	233	*Harmonia axyridis*	1e-92	XP_045468643.1	73.89
*Sex peptide receptor 1*	Csep009323.1	1,023	*Harmonia axyridis*	0.0	XP_045464657.1	91.47
*Tachykinin-like peptides receptor*	Csep027247	191	*Coccinella septempunctata*	7e-30	XP_044762535.1	98.31

ORF: open reading frame.

### 3.3 Phylogenetic analyses

**FIGURE 1:**
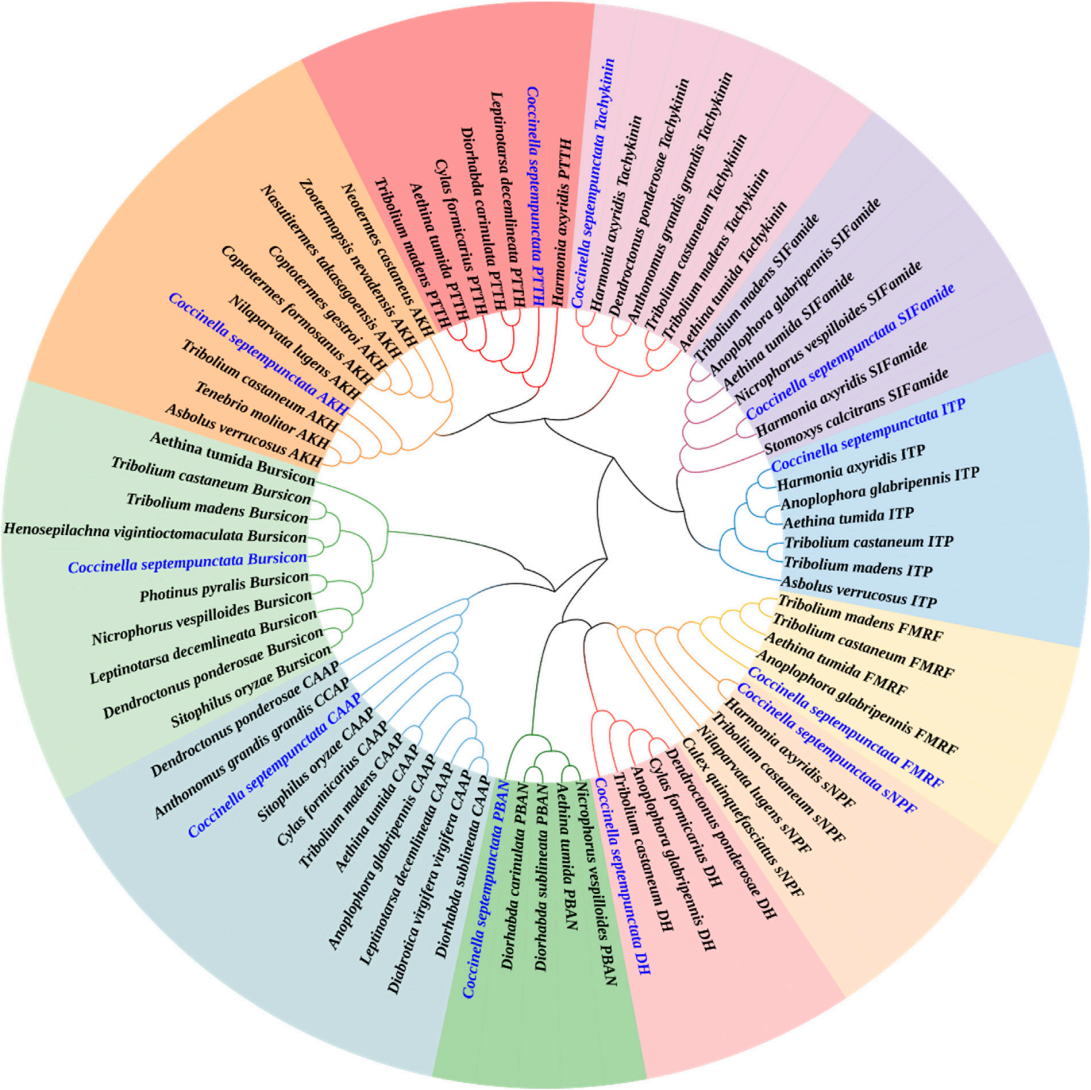
Phylogenetic tree of neuropeptides from *C. septempunctata* and other insects. The *C. septempunctata* neuropeptides are highlighted in blue color, accession numbers are given in Supplementary Table S1. The tree was conducted with MEGA v.11, using the Maximum-Likelihood method and the bootstrap analysis with 1,000 replicates.

**FIGURE 2:**
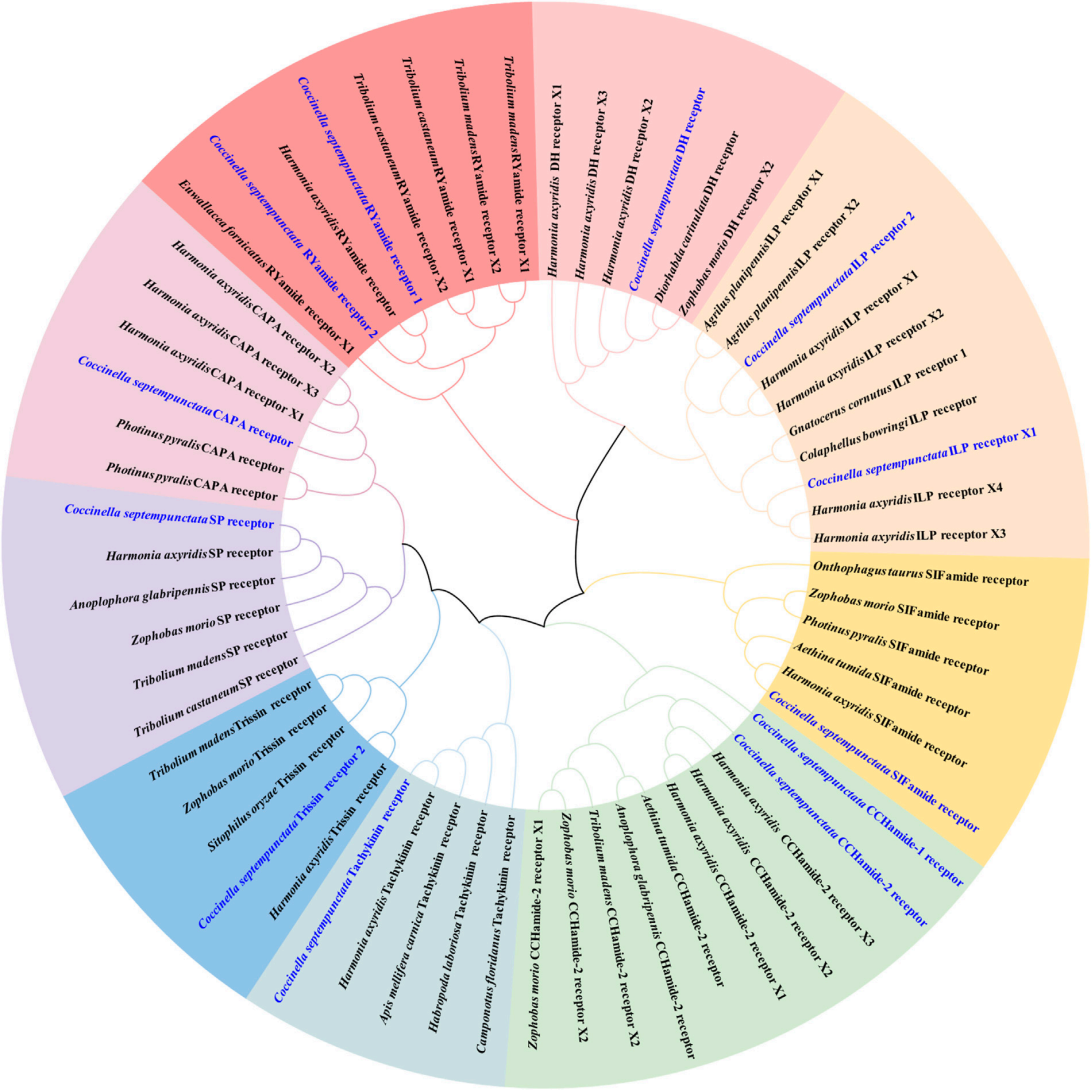
Phylogenetic tree of neuropeptide receptors from *C. septempunctata* and other insects. The *C. septempunctata* neuropeptide receptors are highlighted in blue color, accession numbers are given in Supplementary Table S2. The tree was conducted with MEGA v.11, using the Maximum-Likelihood method and the bootstrap analysis with 1,000 replicates.

Neuropeptide and neuropeptide receptor sequences of *C. septempunctata* were respectively used to construct maximum likelihood phylogenetic trees with 68 published neuropeptide sequences and 50 published neuropeptide receptor sequences from coleoptera, diptera and homoptera (illustrated in Figures 1, 2). Among all neuropeptides, sNPF, FMRF, PBAN, Tachykinin, PTTH, AKH, CAAP, SIFamide, DH, ITP and Bursicon were clustered together with the orthologs from other coleoptera insects in the same clade. And the neuropeptide receptor is also in a similar situation.

### 3.4 Multiple sequence alignment analysis of partial neuropeptides and prediction of mature neuropeptides

**FIGURE 3:**
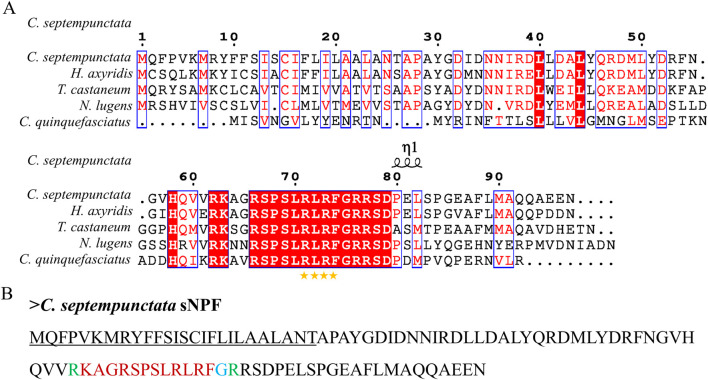
The amino acid sequence alignment of sNPF in *C. septempunctata* **(A)** and other insects and predicted structures of neuropeptide precursors **(B)**. The conservative motif “FLRFamide” is labeled with stars. The signal peptide region is underlined; The area marked with red is the mature peptide region; The putative glycine-derived C-terminal amidation sites in blue; The cleavage site marked with green. Gene Accesion No.: C. s*eptempunctata*:this study, *Harmonia axyridis*: XP_045470701.1, *Tribolium castaneum*: XP_008198705.1, *Culex quinquefasciatus*: EDS32331.1, *Nilaparvata lugens*: XP_022184541.1.

**FIGURE 4:**
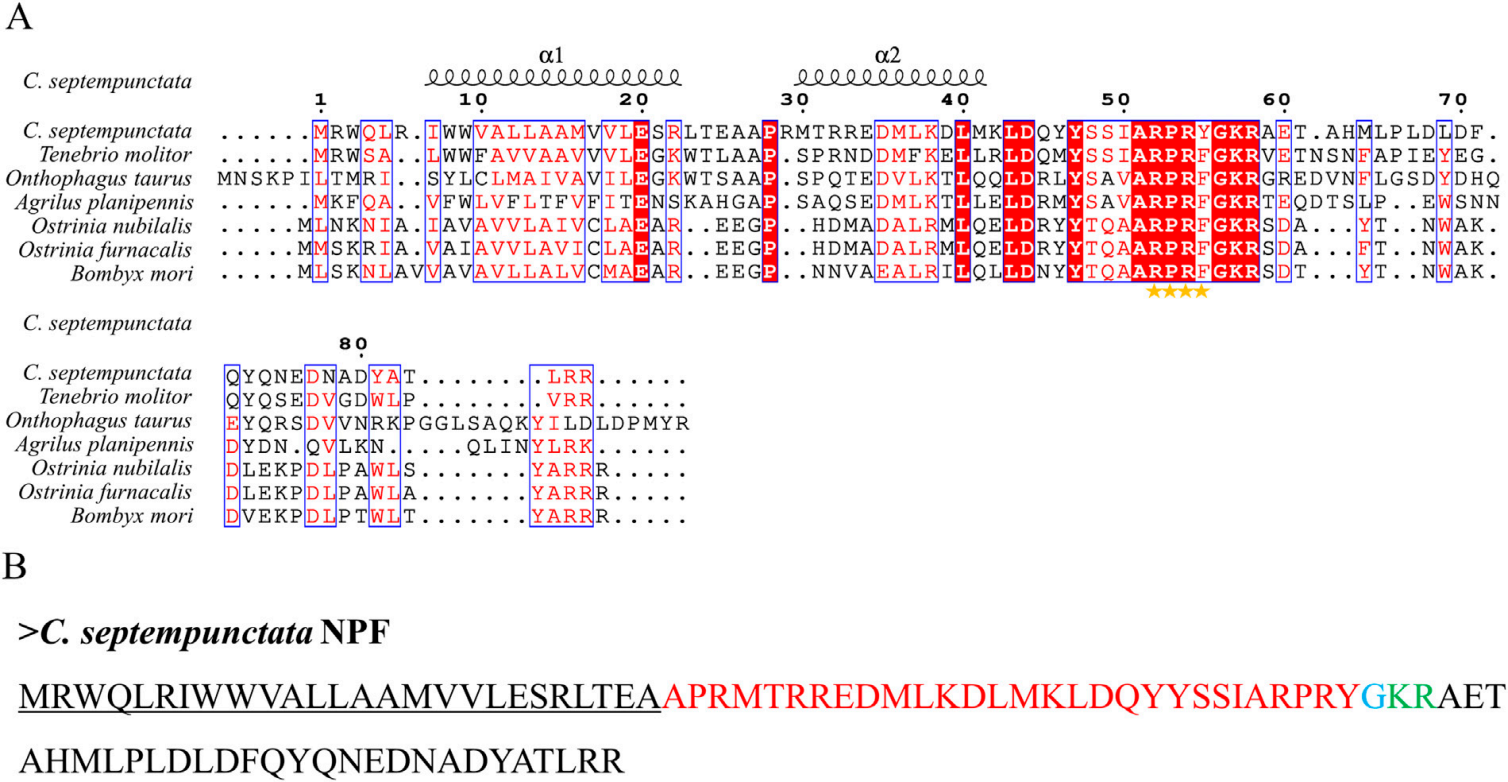
The amino acid sequence alignment of NPF in *C. septempunctata* **(A)** and other insects and predicted mature peptide structure **(B)**. The conservative motif”RPRF/Yamide” is labeled with stars. The signal peptide region is underlined; The area marked with red is the mature peptide region; The putative glycine-derived C-terminal amidation sites in blue; The cleavage site is marked with green.

**FIGURE 5:**
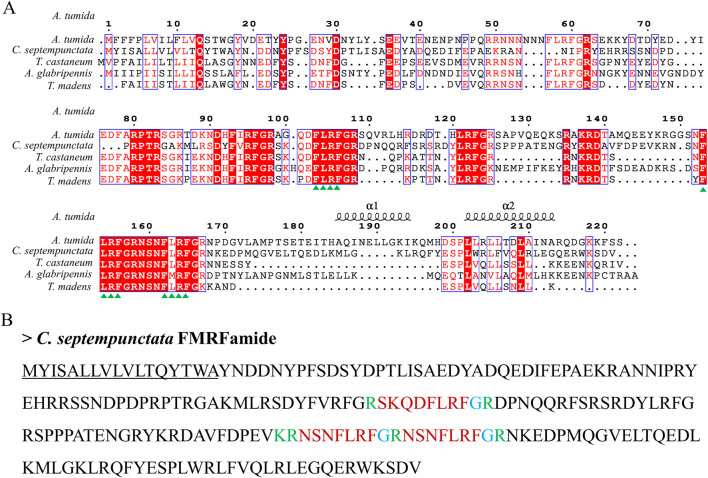
The amino acid sequence alignment of FMRFamide in *C. septempunctata* and other insects **(A)** and predicted mature peptide structure **(B)**. The conservative motif “FLRFamide” are labeled with triangles. The signal peptide region is underlined; The area marked with red is the mature peptide region; The putative glycine-derived C-terminal amidation sites in blue; The cleavage site is marked with green. Gene Accesion No.: *C.* sep*tempunctata:* this study, *Aethina tumida*: XP_019869081.1, *Tribolium castaneum*: XP_008191572.1, *Tribolium madens*: XP_044254202.1, *Anoplophora glabripennis*: XP_023310731.1.

**FIGURE 6:**
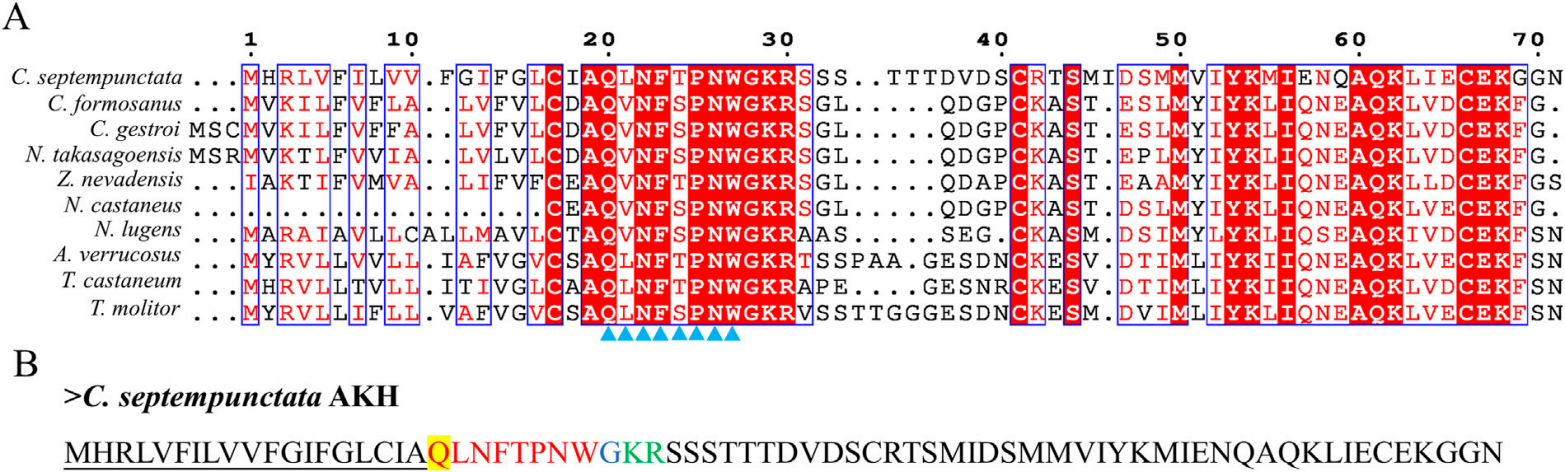
The amino acid sequence alignment of AKH in *C. septempunctata* and other insects **(A)** and predicted mature peptide structure **(B)**The conservative motif “QX_6_W” are labeled with triangles. The signal peptide region is underlined; The phosphorylation sites is highlighted in yellow; The area marked with red is the mature peptide region; shaded in blue is the amide site, The area marked with green is the cleavage site. Gene Accesion No.: *C. septempunctata:*this study, *Coptotermes formosanus*: AML80822.1, *Coptotermes gestroi*: AML80828.1, *Nasutitermes takasagoensis*: AML80829.1, *Zootermopsis nevadensis*: AML80834.1, *Nilaparvata lugens*: AFN26934.1, *Neotermes castaneus*: AML80825.1, *Asbolus verrucosus*: RZB41086.1, *Tribolium castaneum*: NP_001107818.1, *Tenebrio molitor*: UXO98184.1.

**FIGURE 7:**
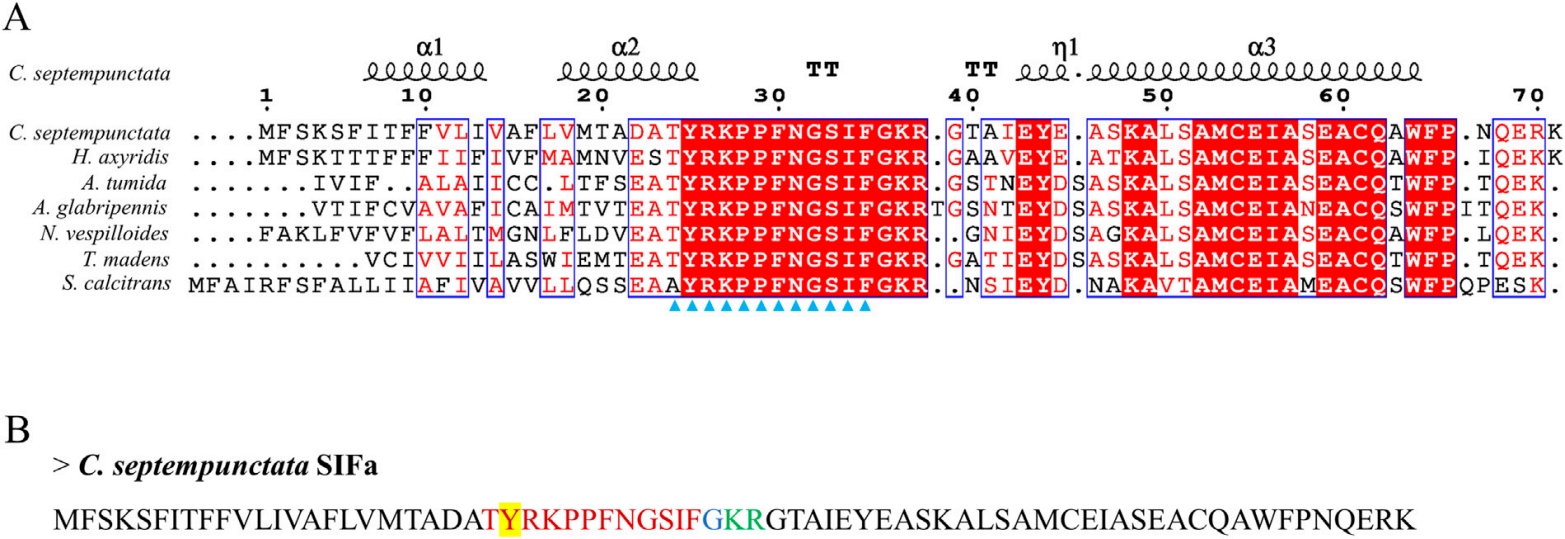
The amino acid sequence alignment of SIFa in *C. septempunctata* and other insects **(A)** and predicted mature peptide structure **(B)**. The conservative motif “XYRKPPFNGSIF” are labeled with triangles. The signal peptide region is underlined; The sulfation of tyrosine residues is highlighted in yellow; The area marked with red is the mature peptide region; shaded in blue is the amide site, The area marked with green is the cleavage site. Gene Accesion No.: C*. septempunctata:*this study, *Harmonia axyridis*: XP_045483513.1, *Tribolium madens*: XP_044265653.1, *Aethina tumida*: XP_019881334.1, *Nicrophorus vespilloides*: XP_017769590.1, *Anoplophora glabripennis*: XP_018576888.1, *Stomoxys calcitrans*: XP_013114449.1.

**FIGURE 8:**
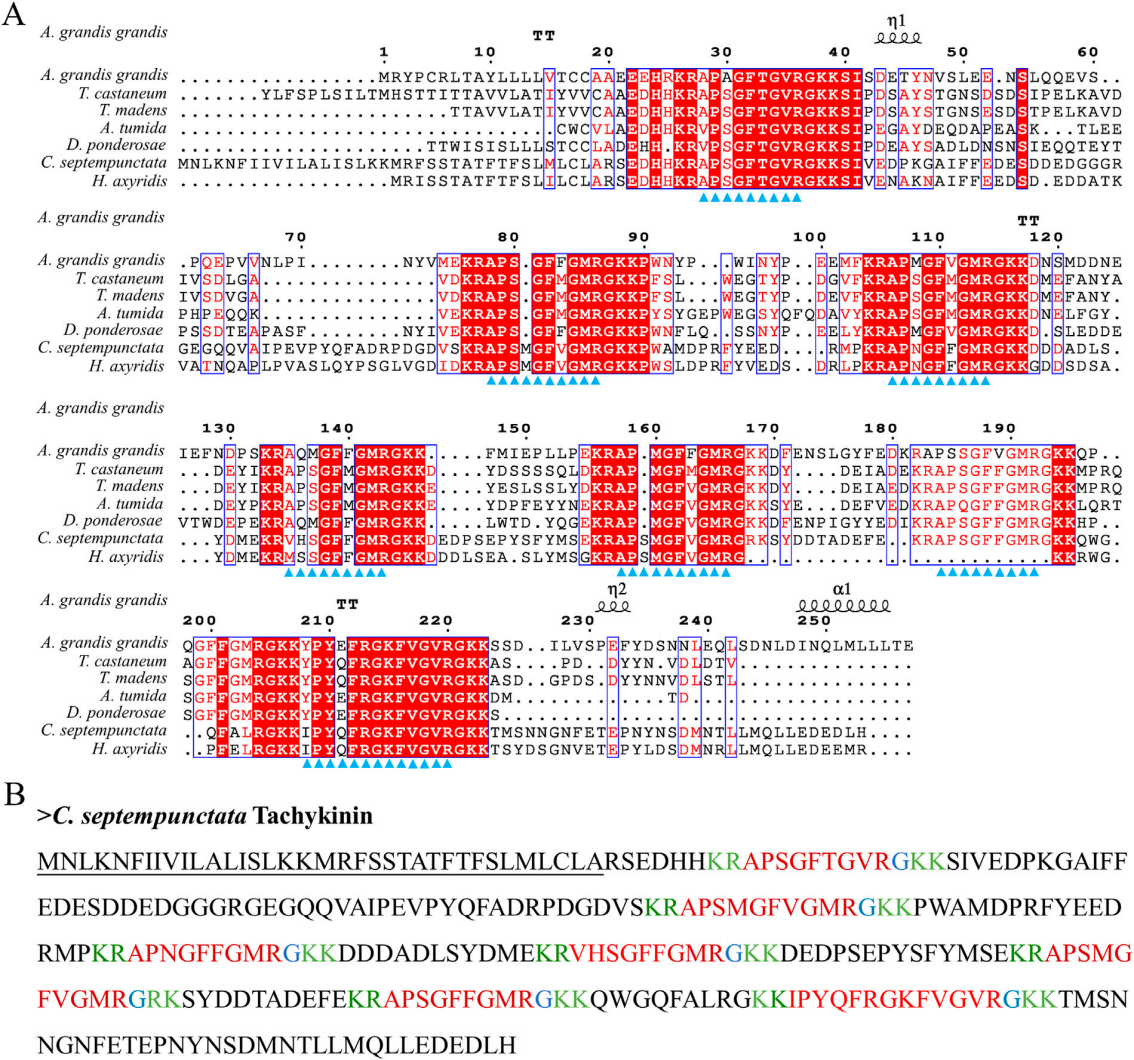
The amino acid sequence alignment of Tachykinin in *C. septempunctata* and other insects **(A)** and predicted mature peptide structure **(B)**. Note: The conservative motif “FXGXR” are labeled with triangles. The signal peptide region is underlined; The area marked with red is the mature peptide region; shaded in blue is the amide site, The area marked with green is the cleavage site. Gene Accesion No.: C. *septempunctata:*this study, *Harmonia axyridis*: XP_045478364.1, *Tribolium castaneum*: KYB25860.1, *Dendroctonus ponderosae*: XP_019770548.1, *Tribolium madens*: XP_044272560.1. *Aethina tumida*: XP_019880636.2, *Anthonomus grandis*: XP_050299773.1.

**FIGURE 9:**
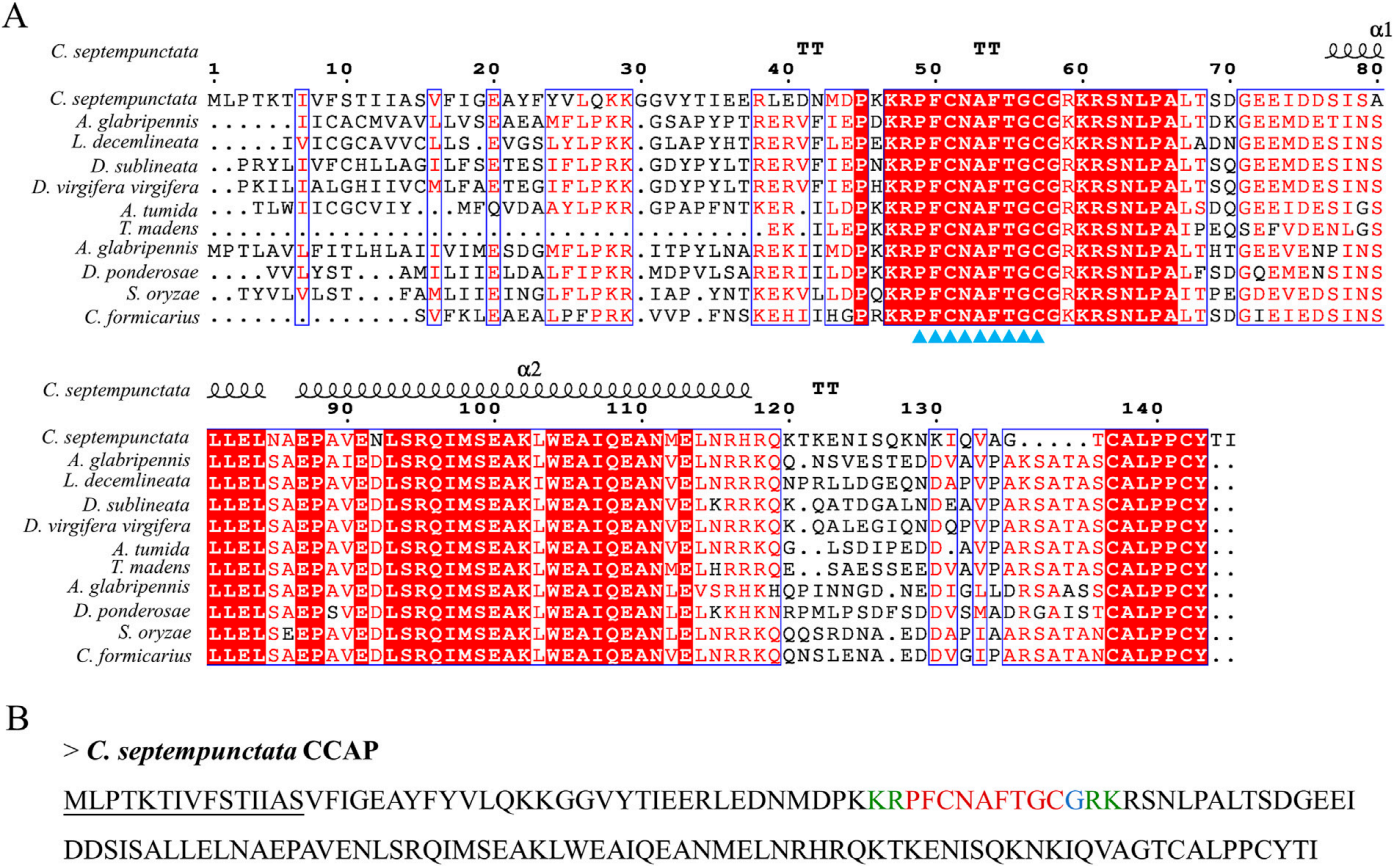
The amino acid sequence alignment of CCAP in *C. septempunctata* and other insects **(A)** and predicted mature peptide structure **(B)**. Note: The conservative motif “PFCNAFTGCamide” is labeled with triangles. The signal peptide region is underlined; The area marked with red is the mature peptide region; shaded in blue is the amide site, The area marked with green is the cleavage site. Gene Accesion No.: C*. septempunctata:*this study, *Diorhabda sublineata*: XP_056637896.1, *Diabrotica virgifera*: XP_028132314.1, *Anoplophora glabripennis*: XP_018571850.1, *Anthonomus grandis*: XP_050299286.1, *Aethina tumida*: XP_019874422.1, *Dendroctonus ponderosae*: XP_019766469.1, *Sitophilus oryzae* XP_030753057.1, *Leptinotarsa decemlineata*: XP_023012719.1, *Cylas formicarius*: XP_060524530.1, *Tribolium madens*: XP_044258689.1.

**FIGURE 10:**
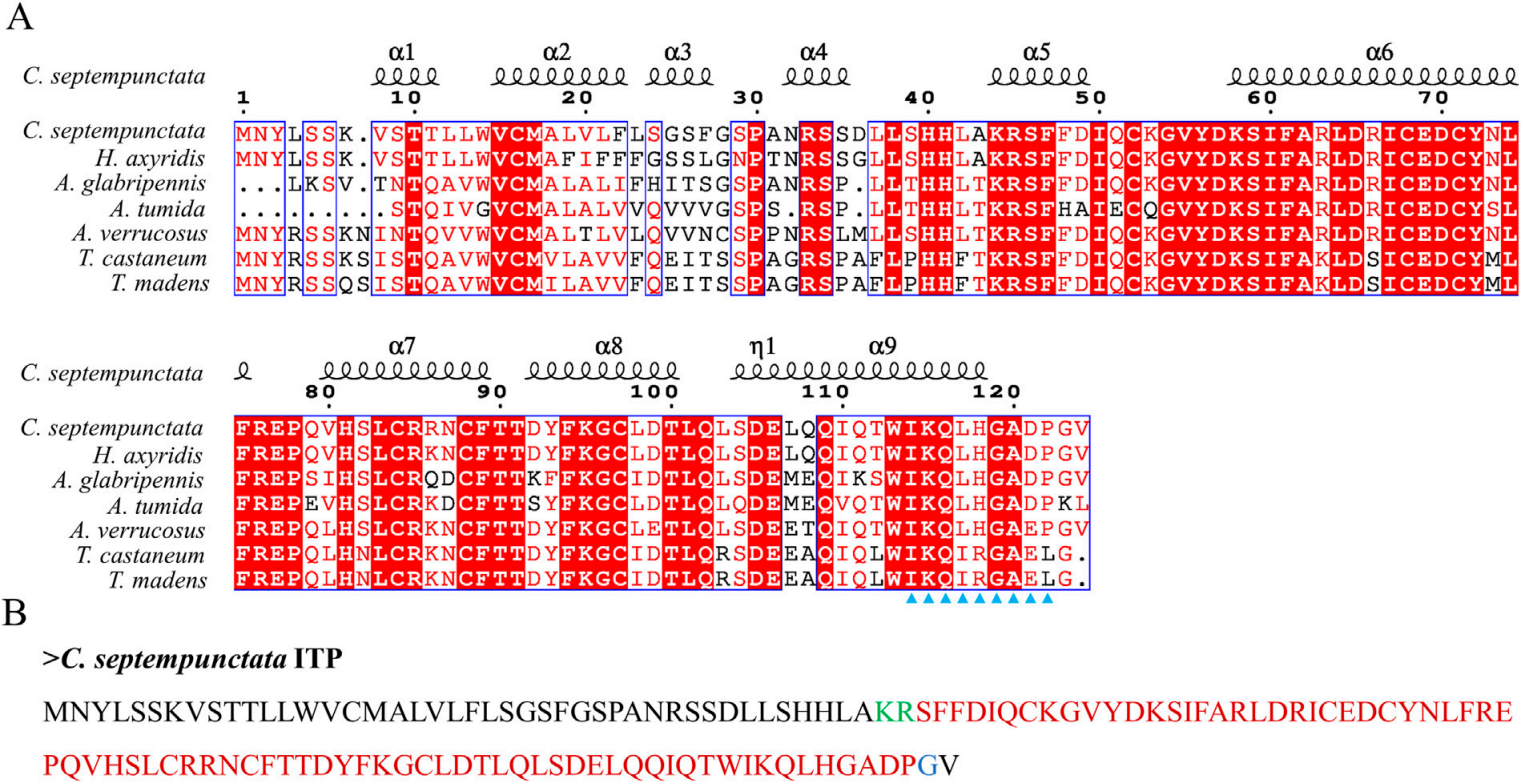
The amino acid sequence alignment of ITP in *C. septempunctata* and other insects **(A)** and predicted mature peptide structure **(B)**. The conservative motif “IKXLHGAXP” are labeled with triangles. The area marked with red is the mature peptide region; shaded in blue is the amide site, The area marked with green is the cleavage site. Gene Accesion No.: C*. septempunctata:*this study, *Harmonia axyridis*: XP_045478547.1, *Asbolus verrucosus*: RZC33292.1: *Anoplophora glabripennis*: XP_018574385.1, *Tribolium castaneum*: XP_008195066.1, *Tribolium madens*: XP_044263512.1, *Aethina tumida*: XP_019869750.1.

By aligning neuropeptides protein sequence with orthologs from different insect species, The signal peptides, mature peptides and conserved motifs were respectively found in sNPF (Figure 3), NPF (Figure 4), FMRFamide (Figure 5), AKH (Figure 6), SIFa (Figure 7), Tachykinin (Figure 8), CCAP (Figure 9) and ITP (Figure 10). Unlike other insects, the C-terminal motif of *C. septempunctata* NPF mature peptide is”RPRYamide”.

### 3.5 Times and tissues expression profile of *C. septempunctata* neuropeptides precursor under diapause inducing condition and normal developmental condition

**FIGURE 11:**
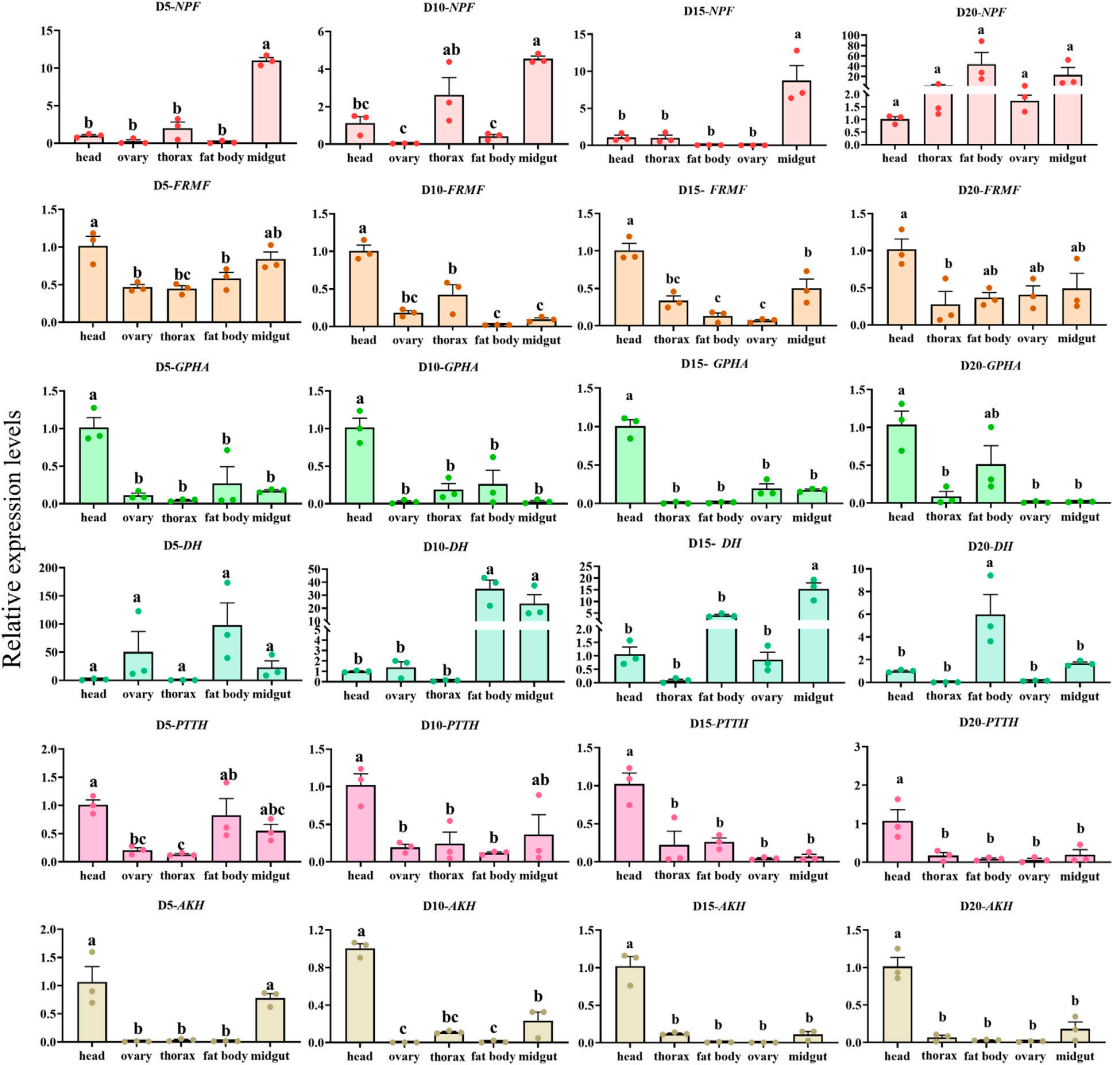
Tissue expression of neuropeptides precursor gene in *C. septempunctata* under the diapause inducing condition. The transcript level was measured via qRT-PCR and normalized against *Actin* gene. Data are presented as the Mean ± SE. The data were statistically analyzed by one-way ANOVA followed by Tukey’s HSD. The letters “a”, “b” and “c”indicate significant difference at the 0.05 level.

**FIGURE 12:**
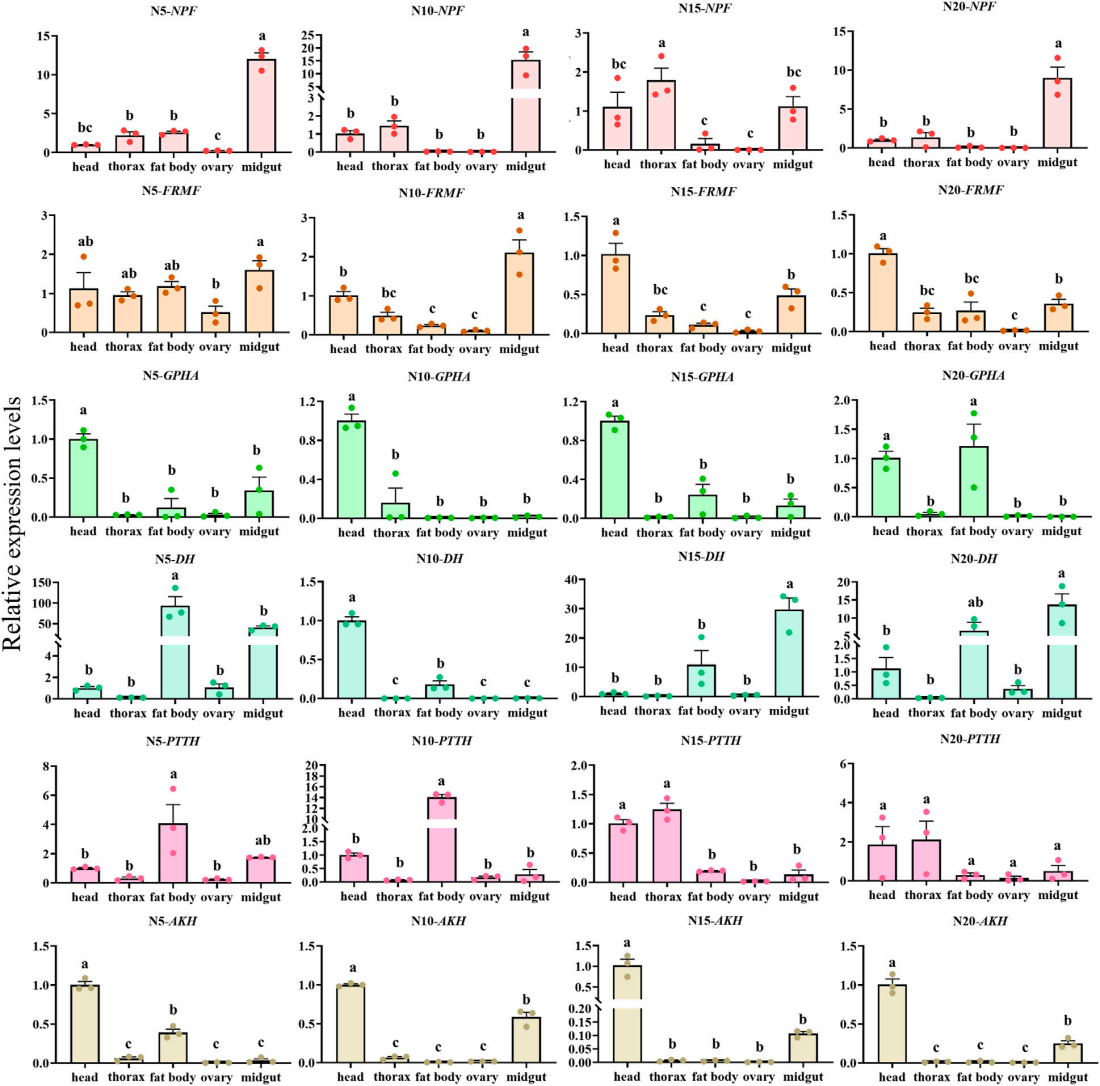
Tissue expression of neuropeptides precursor in *C. septempunctata* under the normal developmental condition. The transcript level was measured via qRT-PCR and normalized against *Actin* gene. Data are presented as the Mean ± SE. The data were statistically analyzed by one-way ANOVA followed by Tukey’s HSD. The letters “a”, “b” and “c”indicate significant difference at the 0.05 level.

**FIGURE 13:**
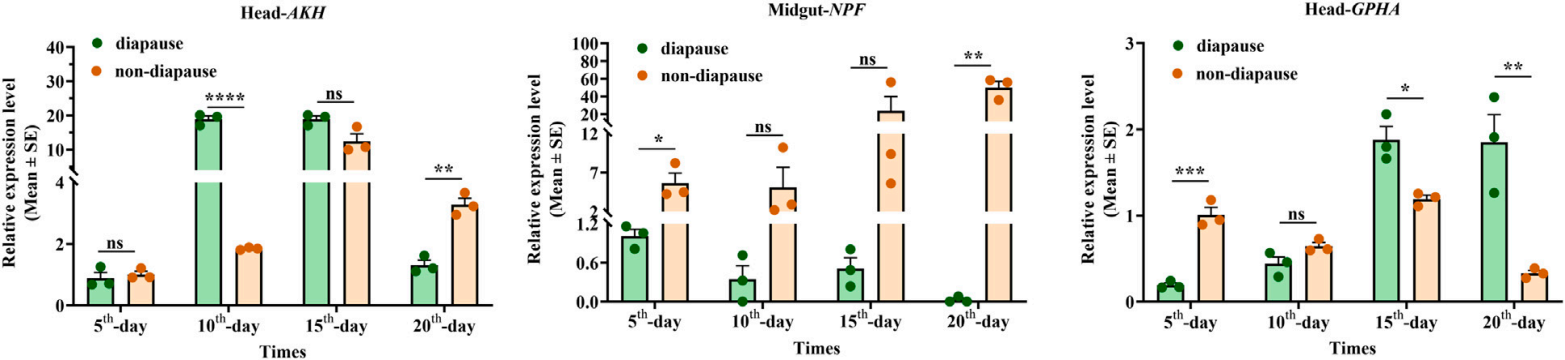
Time expressions of partial neuropeptides precursor in *C. septempunctata* under diapause/non-diapause condition. The mRNA expression levels of *AKH* gene and *GPHA* gene in head and *NPF* gene in midgut were measured at various time points, and data are presented as Mean ± SE of triplicate biological replicates. Asterisk and “ns” indicate significant differences (*t*-test; *p < 0.05, **p < 0.01, ***/****p < 0.001).

The overall relative expression profiles of 6 neuropeptides precursor of *C. septempunctata* in different tissues (head, thorax, fat body, ovary and midgut) of female adults under different inducing conditions were analyzed using RT-qPCR (Figures 11, 12). Under diapause-inducing conditions, neuropeptides precursor *FRMF*, *GPHA*, *AKH*, and *PTTH* exhibited significantly higher expression levels in the head compared to other tissues. *NPF* was predominantly expressed in the midgut of *C. septempunctata* under diapause inducing condition (Figure 11). Under normal developmental condition, *AKH* and *GPHA* showed predominant expression in the head, while *NPF* was highly expressed in the midgut. *FRMF* displayed predominant expression in both the head and midgut (Figure 12). During diapause induction, the mRNA abundance of *AKH* was notably higher on the 10th day compared to non-diapause females, but decreased by the 20th day. In contrast, *GPHA* showed lower expression levels on the 5th day of diapause induction compared to non-diapause females, but increased significantly by the 15th and 20th days. Interestingly, throughout the diapause induction stages, *NPF* consistently showed lower expression levels compared to non-diapause females (Figure 13).

## 4 Discussion

Transcriptome sequencing is fundamental to dentification of genes, and identification of neuropeptides and their receptors is the first and foremost step of deep function depth studies in physiological processes. However, detailed information on neuropeptide types and expression in *C. septempunctata* remains scarce. Therefore, to address this problem, we performed mRNA sequencing analysis of the head in *C. septempunctata*.

Based on transcriptome analysis, we identified 17 neuropeptides precursor and 12 receptor transcripts encoding 9 neuropeptide receptors from *C. septempunctata* in this study. The confirmation of these receptor information still requires a lot of work, such as pharmacological characterization was determined with all possible neuropeptides of similar motifs. Most neuropeptide receptors belong to the G protein-coupled receptor family. However, we have noticed that insulin-like receptor isoform XI and insulin-like receptor are not GPCRs but tyrosine kinase receptors (TKR), which includes catalytic domain of insulin receptor-like protein tyrosine kinases, furin-like cysteine rich region, receptor l domain and fibronectin type domain (Choi and Bai, 2023). For some neuropeptide signaling systems, *C. septempunctata* seems to lack both receptor and neuropeptide precursor, such as SK, Crz, Trissin, Ast and Elevenin. For others, the neuropeptide precursor was not found or is lacking, but its presumed receptor is present (e.g., PDF, ILP, CNMa, etc.). There are several factors that may account for the difference in the number of identified genes of specific functions which has been discussed (Wang et al., 2015; Huang et al., 2021; Li X. et al., 2020). Firstly, the head used as the sequenced samples did not cover complete the individual and all stages of life cycle of *C. septempunctata* and some genes may not have been expressed in these measured samples. Secondly, some genes are expressed at low levels in head tissue making it challenging to detect their expression quantitatively or they may not be expressed at all in the sampled conditions. Thirdly, due to the lack of strong sequence conservation among some neuropeptide precursors or neuropeptide receptors, their clear orthologs could not be found in *C. septempunctata* based on homology searches. Lastly, some neuropeptides may indeed be absent in *C. septempunctata*, a phenomenon observed in various insects. For instance, sex peptide was reported only in the *Drosophila genus*, while neuroparsin is missing in *Drosophila melanogaster* and other *Drosophila* species (Veenstra, 2010).

Generally, neuropeptide precursor undergoes alternative splicing, regulated cleavages and post-translational to produce functionally active mature neuropeptides (Agrawal et al., 2019; Christie, 2017). Multiple sequence alignment of NPF precursors between the *C. septempunctata* and other insects revealed a conserved C-terminal sequence-RPRYamide. The invertebrate NPF is considered to be a superfamily of NPY in which an amidated tyrosine (Y) residue is replaced with an amidated phenylalanine (F) residue at its C-terminal end (Cui and Zhao, 2020). In insects, most NPFs are consisted of 36–40 amino acids and the sequence of amino acids at C-terminal end is relatively conservative with RPRFamide, which is important to maintain functional activity of NPFs. According to the different C-terminus, insect NPFs can be divided into the following type: the general structure of NPFs is RPRFa, which accounts for more than 80% of known insect NPFs (except *drosophila melanogaster* NPF: RVRFa (Brown et al., 1999)); Additionally, a unique structure, RGRYa (often GRxRYa or Gx1x2RYa, NPF2 or NPY), has been identified exclusively in Lepidoptera insects with some species exhibiting both RPRFa and RGRYa structures (Wang et al., 2021; Li et al., 2023); Furthermore, a distinct structure known as KARYa has been found only in Hymenoptera insects, such as *A. mellifera* NPF (Hummon et al., 2006).

NPFs are widely distributed in brain neurons of larvae and adults, as well as the foregut, midgut, and hindgut in most insects (Wang et al., 2021; Li et al., 2023; Johard et al., 2008; Liu et al., 2013; Nässel et al., 2008; Yue et al., 2016). NPF peptides are a group of multifunctional peptides and exert multi-actions in insects. The differential distribution of NPF across various tissues and cells suggests a close relationship between its location and function. For instance, NPF expression in the midgut correlates with feeding behavior, while its expression in the brain (clock neurons, mushroom body) relates to daily circadian rhythm and sleep (Cui and Zhao, 2020). In our study, we observed predominant expression of NPF in the head and midgut, with lower levels in the thorax and fat body of female *C. septempunctata* adults. Interestingly, throughout the entire stage of diapause induction, *NPF* expression levels were consistently lower compared to females under non-diapause conditions. In our previous research, we noted that female *C. septempunctata* exhibit suppressed ovaries and accumulate lipids during prolonged diapause periods, often accompanied by reduced or no feeding (Li FF. et al., 2020; Chen et al., 2023). Given the multifaceted roles of *NPF* in insects and its expression profile in *C. septempunctata*, we plan to conduct further investigations into the functions of *NPF* genes during diapause.

The prothoracicotropic hormone (PTTH) is a brain neurosecretory peptide of insects that stimulates the prothoracic glands (PGs) to secrete ecdysteroid, thereby inducing molting and metamorphosis (Mizoguchi, 2001). It is widely accepted that PTTH is released at specific times during insect development, such as larval molting, larval–pupal transformation and adult development. In addition, Some studies indicate that JH regulates the brain to produce and/or release PTTH. In lepidopterans, JH blocked the PTTH secretion activity of the brain–corpora cardiaca–corpora allata complex (Br–CC–CA) of JHA-treated larvae, thereby indirectly inhibiting the secretion of ecdysteroid that induces the larval–pupal transformation (Rountree and Bollenbacher, 1986; Watson et al., 1987). Our previous research found that the absence of the juvenile hormone (JH) which resulted from the upregulation of two JH degradation genes (*CsJHE*, *CsJHEH*), is a critical factor during reproductive diapause in *C. septempunctata* (Li et al., 2022). It is unknown whether JH regulates the PTTH of *C.septenunctata* females. Further in-depth research is needed to determine whether PTTH is involved in regulating the reproductive diapause of *C. septempunctata* females. Pupal diapause of lepidoptera insects is regulated by the major developmental hormones prothoracicotropic hormone (PTTH) and ecdysone, such as *Scrobipalpa ocellatella* (Ahmadi et al., 2021), *Pieris napi* (Süess et al., 2022) and *Helicoverpa armigera* (Wei et al., 2005). There is currently no research on the regulation of diapause by PTTH in coleoptera insects.

Adipokinetic hormone (AKH) produced by the corpora cardiaca is involved in innutrient and energy metabolism, maintaining lipid, carbohydrate levels and female reproduction in insect (Wei et al., 2005; Lee and Park, 2004; Bharucha et al., 2008). In *D. melanogaster*, decreased AKH reduces trehalose content in hemolymph, glucose homeostasis and low energy metabolism (Lee and Park, 2004; Kim and Rulifson, 2004; Isabel et al., 2005). In *B. mori*, injection of AKH maturation peptide increases carbohydrate content and lipid levels in hemolymph (Sehadova et al., 2020). Knockdown AKHR suppresses the mobilization of triacylglycerol and trehalose, reduces *vitellogenin* expression in the fat body, and delays oocyte development in migratory locusts (Zheng et al., 2020). In *Bactrocera dorsalis*, AKHR silencing causes triglyceride (TG) accumulation and affects courtship activity and fecundity (Hou et al., 2017). In our previous research, *CsAKH* and *CsAKHR* are involved in the regulation of lipid accumulation and ovarian development during diapause in *C. septempunctata* (Shen et al., 2024).

In addition, for the identified neuropeptides and neuropeptide receptors, we conducted evolutionary tree analysis and multiple sequence alignment. Amino acid sequence alignment results showed that CAPA receptor and SP receptor in *C. septempunctata* respectively had a much longer sequence than their closely related species (Supplementary Figures S8 and S9); SIFamide receptor in *C. septempunctata* had a shorter sequence than its closely related species (Supplementary Figure S3). In addition, Supplementary Figure S2 showed that tachykinin receptor in *Harmonia axyridis* had a longer sequence than its closely related species. We infered that this may be caused by alternative splicing. Due to selection pressure or optimization of gene expression, the alternative splicing of precursor mRNA leads to additional exon insertion or intron retention, resulting in the production of various mature mRNA molecules (Koch, 2017). These different mRNA have distinct functional or structural features. So, genes of certain species may have shorter or longer mRNA sequences than homologous genes of other species.

## 5 Conclusion

In conclusion, our study employed transcriptome sequencing to identify a comprehensive panel of 17 neuropeptides precursor and 9 neuropeptide receptors genes from the adult head of *C. septempunctata*. This initial exploration provides a foundational understanding of these genes’ functions. We conducted phylogenetic analyses with sequences of other insect species and examined their gene expression profiles in different tissues including the head, thorax, fat body, ovary and midgut in female. These findings suggest their potential roles in the development and diapause of *C. septempunctata*. Overall, our research contributes valuable insights into neuropeptides precursor and neuropeptide receptor genes in insect diapause, advancing our understanding and potentially facilitating the mass production and storage of *C. septempunctata*.

## Data Availability

The data presented in the study are deposited in the NCBI repository: https://www.ncbi.nlm.nih.gov/, accession number PRJNA1154946.
