# Supplementary material for: Identification and expression profiling of neuropeptides and neuropeptide receptor genes in a natural enemy, Coccinella septempunctata
Source: Front Physiol. 2024 Oct 9;15:1464989. doi: 10.3389/fphys.2024.1464989 (PMC11496152; doi:10.3389/fphys.2024.1464989)
Supplement: Supplementary file 1 [file Table1.DOCX]

Table S1. Information on the insects that are included in the phylogenetic analysis.

| **Order** | **Species** | **GenBank No.** |
| --- | --- | --- |
| **sNPF** | *Coccinella septempunctata* | This study |
|  | *Harmonia axyridis* | XP_045470701.1 |
|  | *Tribolium castaneum* | XP_008198705.1 |
|  | *Culex quinquefasciatus* | EDS32331.1 |
|  | *Nilaparvata lugens* | XP_022184541.1 |
| **FMRF** | *Coccinella septempunctata* | This study |
|  | *Aethina tumida* | XP_019869081.1 |
|  | *Anoplophora glabripennis* | XP_023310731.1 |
|  | *Tribolium madens* | XP_044254202.1 |
|  | *Tribolium castaneum* | XP_008191572.1 |
| **PBAN** | *Coccinella septempunctata* | This study |
|  | *Nicrophorus vespilloides* | XP_017786058.1 |
|  | *Diorhabda sublineata* | XP_056645980.1 |
|  | *Diorhabda carinulata* | XP_057657574.1 |
|  | *Aethina tumida* | XP_019871287.1 |
| **Tachykinin** | *Coccinella septempunctata* | This study |
|  | *Harmonia axyridis* | XP_045478364.1 |
|  | *Tribolium castaneum* | KYB25860.1 |
|  | *Dendroctonus ponderosae* | XP_019770548.1 |
|  | *Tribolium madens* | XP_044272560.1 |
|  | *Aethina tumida* | XP_019880636.2 |
|  | *Anthonomus grandis grandis* | XP_050299773.1 |
| **PTTH** | *Coccinella septempunctata* | This study |
|  | *Harmonia axyridis* | XP_045479904.1 |
|  | *Tribolium madens* | XP_044254998.1 |
|  | *Cylas formicarius* | XP_060531974.1 |
|  | *Diorhabda carinulata* | XP_057655514.1 |
|  | *Leptinotarsa decemlineata* | XP_023026695.1 |
|  | *Aethina tumida* | XP_019869567.2 |
| **AKH** | *Coccinella septempunctata* | This study |
|  | *Asbolus verrucosus* | RZB41086.1 |
|  | *Tenebrio molitor* | UXO98184.1 |
|  | *Tribolium castaneum* | NP_001107818.1 |
|  | *Nilaparvata lugens* | AFN26934.1 |
|  | *Coptotermes formosanus* | AML80822.1 |
|  | *Coptotermes gestroi* | AML80828.1 |
|  | *Zootermopsis nevadensis* | AML80834.1 |
|  | *Neotermes castaneus* | AML80825.1 |
|  | *Nasutitermes takasagoensis* | AML80829.1 |
| **CCAP** | *Coccinella septempunctata* | This study |
|  | *Diorhabda sublineata* | XP_056637896.1 |
|  | *Diabrotica virgifera virgifera* | XP_028132314.1 |
|  | *Anoplophora glabripennis* | XP_018571850.1 |
|  | *Anthonomus grandis grandis* | XP_050299286.1 |
|  | *Aethina tumida* | XP_019874422.1 |
|  | *Dendroctonus ponderosae* | XP_019766469.1 |
|  | *Sitophilus oryzae* | XP_030753057.1 |
|  | *Leptinotarsa decemlineata* | XP_023012719.1 |
|  | *Cylas formicarius* | XP_060524530.1 |
|  | *Tribolium madens* | XP_044258689.1 |
| **SIFamide** | *Coccinella septempunctata* | This study |
|  | *Harmonia axyridis* | XP_045483513.1 |
|  | *Tribolium madens* | XP_044265653.1 |
|  | *Aethina tumida* | XP_019881334.1 |
|  | *Nicrophorus vespilloides* | XP_017769590.1 |
|  | *Anoplophora glabripennis* | XP_018576888.1 |
|  | *Stomoxys calcitrans* | XP_013114449.1 |
| **DH** | *Coccinella septempunctata* | This study |
|  | *Tribolium castaneum* | NP_001164096.1 |
|  | *Dendroctonus ponderosae* | XP_048520904.1 |
|  | *Anoplophora glabripennis* | XP_018579740.1 |
|  | *Cylas formicarius* | XP_060518868.1 |
| **ITP** | *Coccinella septempunctata* | This study |
|  | *Harmonia axyridis* | XP_045478547.1 |
|  | *Asbolus verrucosus* | RZC33292.1 |
|  | *Anoplophora glabripennis* | XP_018574385.1 |
|  | *Tribolium castaneum* | XP_044263512.1 |
|  | *Aethina tumida* | XP_019869750.1 |
|  | *Tribolium madens* | XP_044263512.1 |
| **Bursicon** | *Coccinella septempunctata* | This study |
|  | *Henosepilachna vigintioctomaculata* | KAK9888420.1 |
|  | *Aethina tumida* | XP_019870760.1 |
|  | *Sitophilus oryzae* | XP_030754853.1 |
|  | *Leptinotarsa decemlineata* | XP_023014168.1 |
|  | *Tribolium madens* | XP_044267372.1 |
|  | *Tribolium castaneum* | NP_001107779.1 |
|  | *Nicrophorus vespilloides* | XP_017771075.1 |
|  | *Dendroctonus ponderosae* | XP_019755391.2 |
|  | *Photinus pyralis* | XP_031343673.1 |

Table S2. Information on the insects that are included in the phylogenetic analysis and amino acid sequence alignment.

| **Order** | **Species** | **GenBank No.** |
| --- | --- | --- |
| **CAPA receptor** | *Coccinella septempunctata* | This study |
|  | *Harmonia axyridis* | XP_045464272.1 |
|  | *Harmonia axyridis* | XP_045464273.1 |
|  | *Harmonia axyridis* | XP_045464274.1 |
|  | *Photinus pyralis* | XP_031346676.1 |
|  | *Photinus pyralis* | XP_031347093.1 |
| **SP receptor** | *Coccinella septempunctata* | This study |
|  | *Harmonia axyridis* | XP_045464657.1 |
|  | *Zophobas morio* | XP_063920165.1 |
|  | *Tribolium madens* | XP_044269980.1 |
|  | *Tribolium castaneum* | NP_001106940.1 |
|  | *Anoplophora glabripennis* | XP_018562967.1 |
| **Trissin receptor** | *Coccinella septempunctata* | This study |
|  | *Harmonia axyridis* | XP_045470325.1 |
|  | *Sitophilus oryzae* | XP_030767837.1 |
|  | *Tribolium madens* | XP_044268302.1 |
|  | *Zophobas morio* | XP_063918045.1 |
| **Tachykinin receptor** | *Coccinella septempunctata* | This study |
|  | *Harmonia axyridis* | XP_045475839.1 |
|  | *Camponotus floridanus* | EFN69306.1 |
|  | *Apis mellifera carnica* | KAG9428468.1 |
|  | *Habropoda laboriosa* | KOC65718.1 |
| **CCHamide receptor** | *Coccinella septempunctata* | This study |
|  | *Coccinella septempunctata* | This study |
|  | *Harmonia axyridis* | XP_045476917.1 |
|  | *Harmonia axyridis* | XP_045476918.1 |
|  | *Harmonia axyridis* | XP_045476923.1 |
|  | *Anoplophora glabripennis* | XP_023313148.1 |
|  | *Aethina tumida* | XP_049817471.1 |
|  | *Zophobas morio* | XP_063916239.1 |
|  | *Zophobas morio* | XP_063916241.1 |
|  | *Tribolium madens* | XP_044252528.1 |
| **SIFamide** **receptor** | *Coccinella septempunctata* | This study |
|  | *Harmonia axyridis* | XP_045465407.1 |
|  | *Onthophagus taurus* | XP_022919670.1 |
|  | *Photinus pyralis* | XP_031335264.1 |
|  | *Aethina tumida* | XP_049820106.1 |
|  | *Zophobas morio* | XP_063906474.1 |
| **ILP receptor** | *Coccinella septempunctata* | This study |
|  | *Coccinella septempunctata* | This study |
|  | *Harmonia axyridis* | XP_045479040.1 |
|  | *Harmonia axyridis* | XP_045479042.1 |
|  | *Harmonia axyridis* | XP_045482517.1 |
|  | *Harmonia axyridis* | XP_045482518.1 |
|  | *Agrilus planipennis* | XP_018329959.1 |
|  | *Agrilus planipennis* | XP_018329960.1 |
|  | *Colaphellus bowringi* | WML96173.1 |
|  | *Gnatocerus cornutus* | BBI47313.1 |
| **DH receptor** | *Coccinella septempunctata* | This study |
|  | *Harmonia axyridis* | XP_045474413.1 |
|  | *Harmonia axyridis* | XP_045474415.1 |
|  | *Harmonia axyridis* | XP_045474416.1 |
|  | *Zophobas morio* | XP_063931808.1 |
|  | *Diorhabda carinulata* | XP_057668155.1 |
| **RYamide receptor** | *Coccinella septempunctata* | This study |
|  | *Coccinella septempunctata* | This study |
|  | *Harmonia axyridis* | XP_045468643.1 |
|  | *Tribolium madens* | XP_044259449.1 |
|  | *Tribolium madens* | XP_044259454.1 |
|  | *Euwallacea fornicatus* | XP_066147090.1 |
|  | *Tribolium castaneum* | XP_008201193.2 |
|  | *Tribolium castaneum* | XP_064212083.1 |


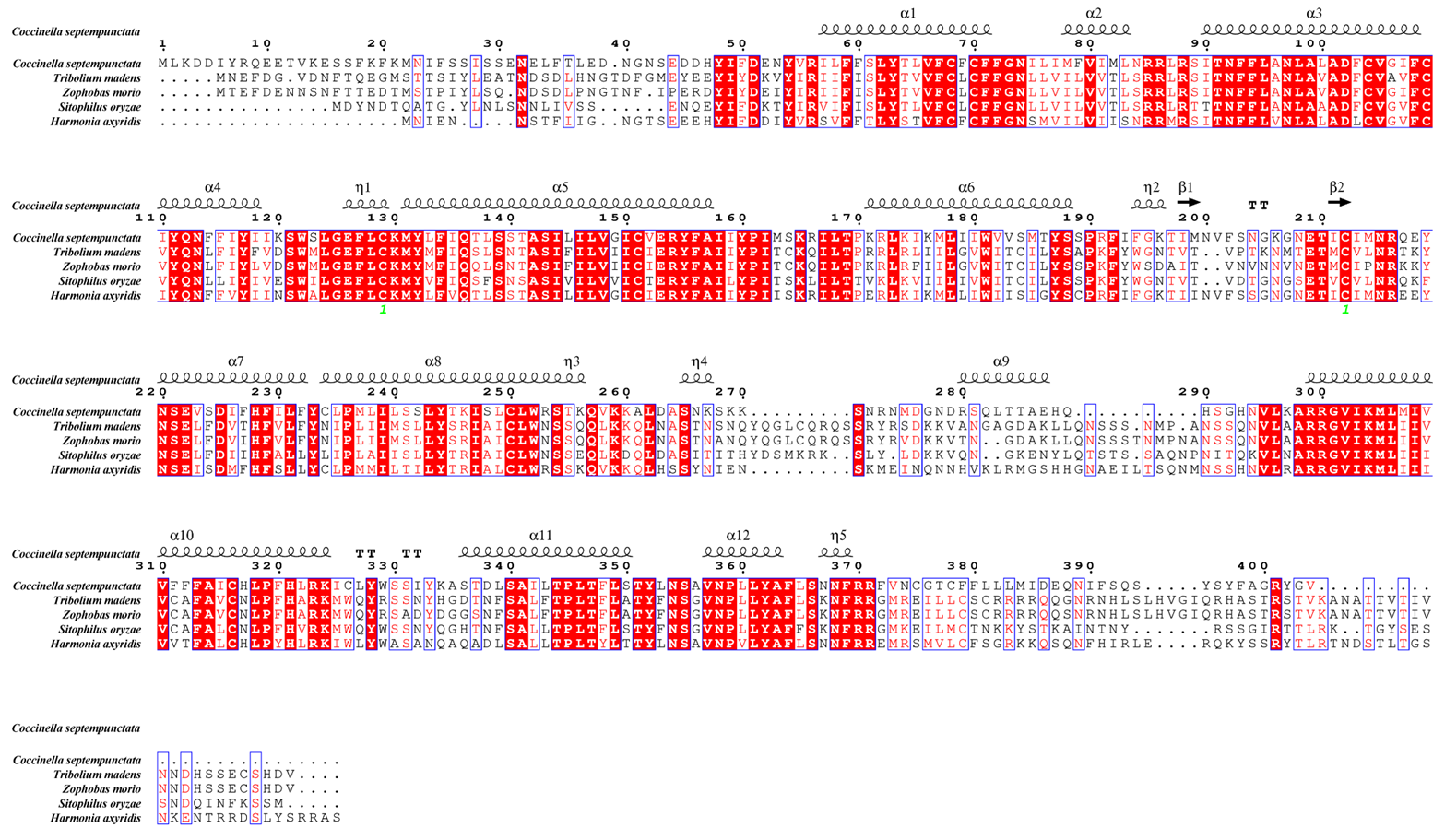


**Figure S1：** **The amino acid sequence alignment of** **trissin receptor in *C. septempunctata***


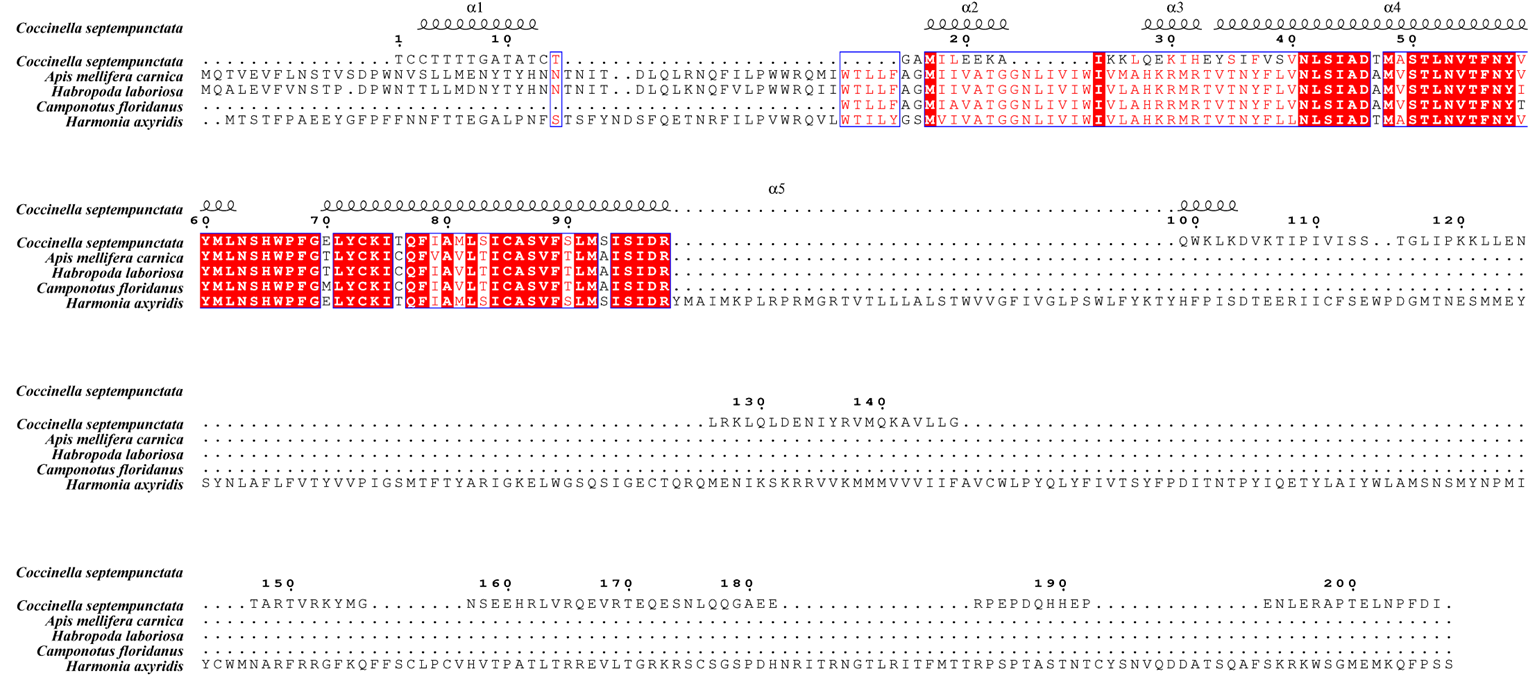


**Figure S2：** **The amino acid sequence alignment of** **tachykinin receptor in *C. septempunctata***


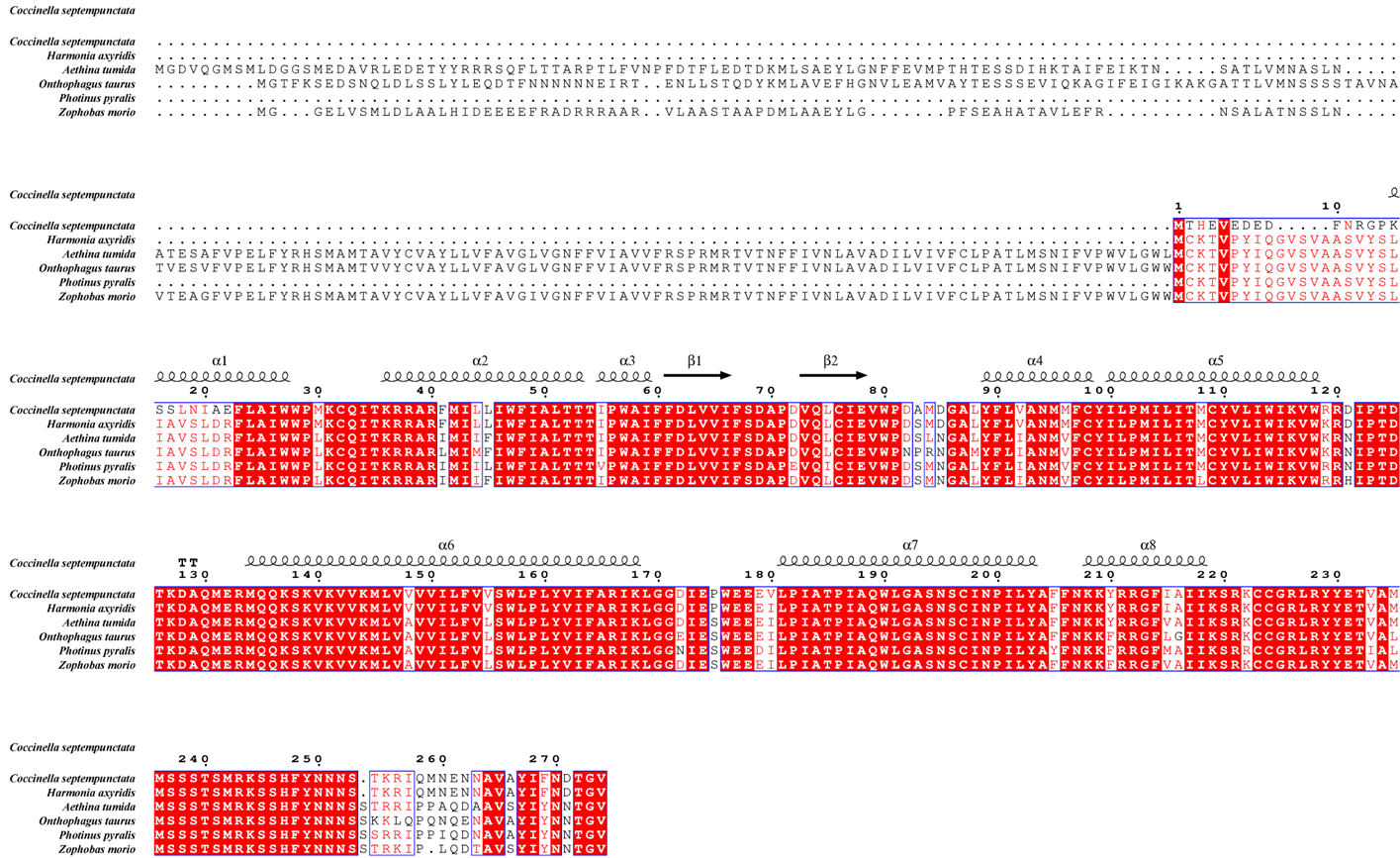


**Figure S3：** **The amino acid sequence alignment of** **SIFamide receptor in *C. septempunctata***

**
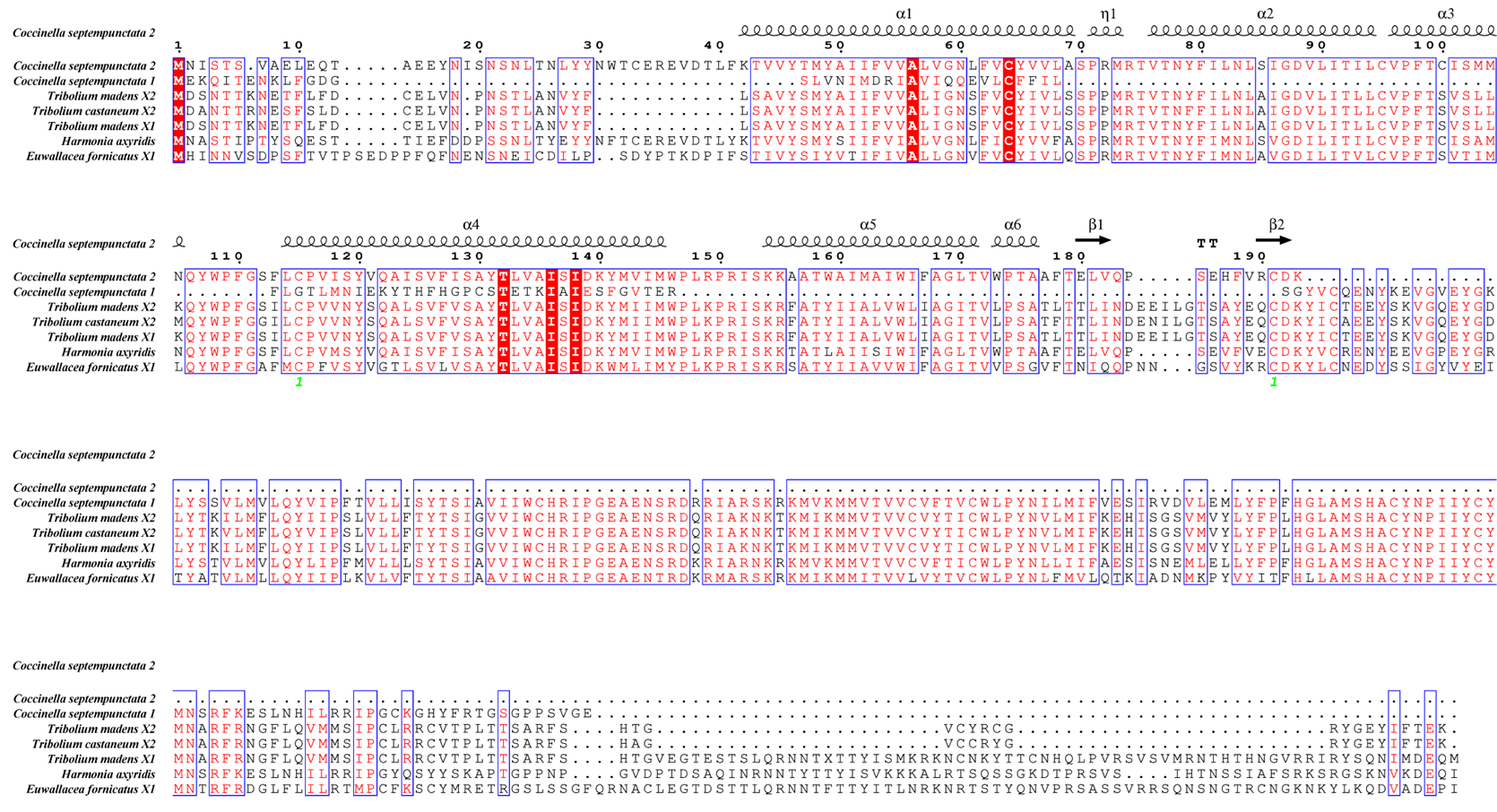
**

**Figure S4：** **The amino acid sequence alignment of** **RYamide receptor in *C. septempunctata***


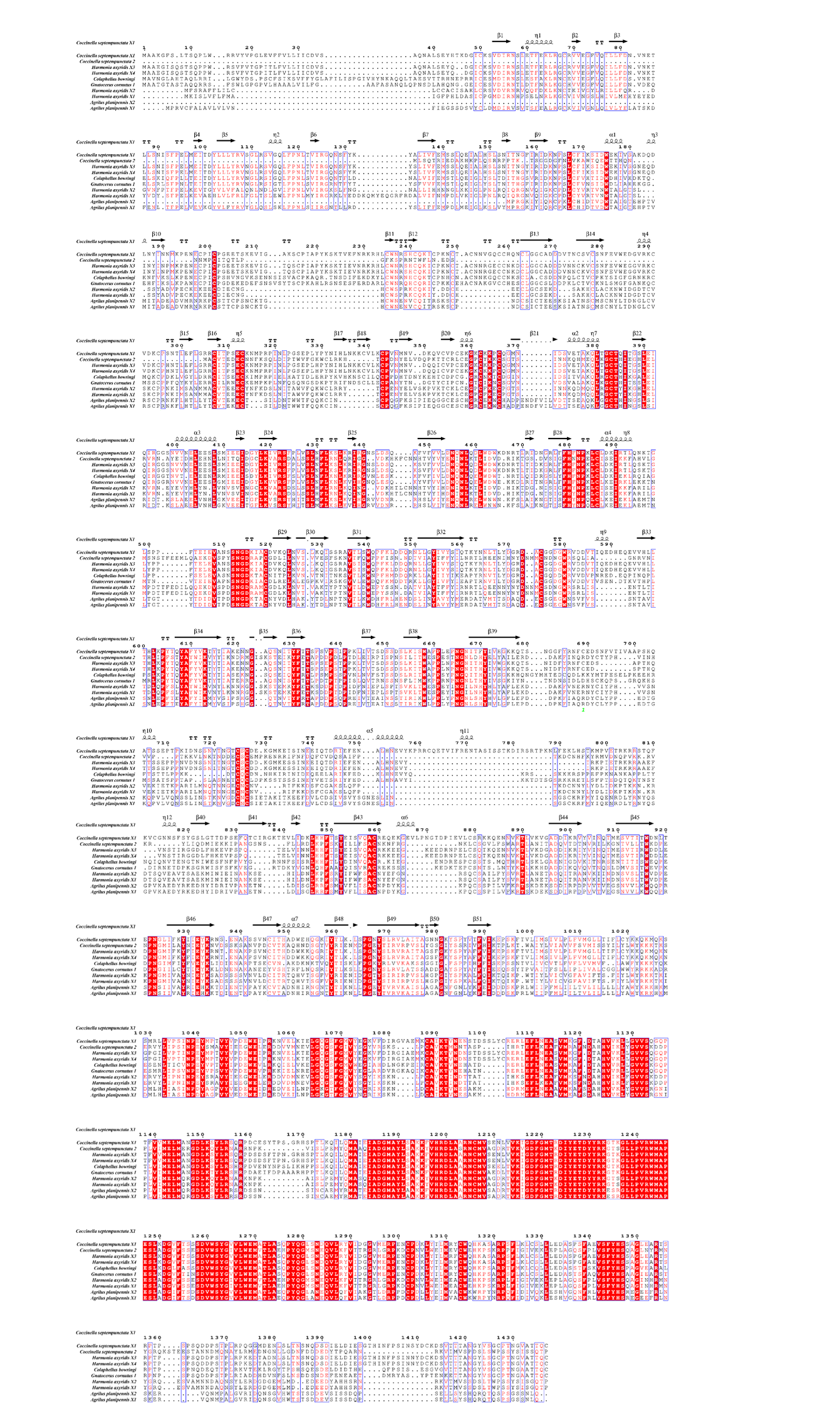


**Figure S5：** **The amino acid sequence alignment of** **ILP receptor in *C. septempunctata***


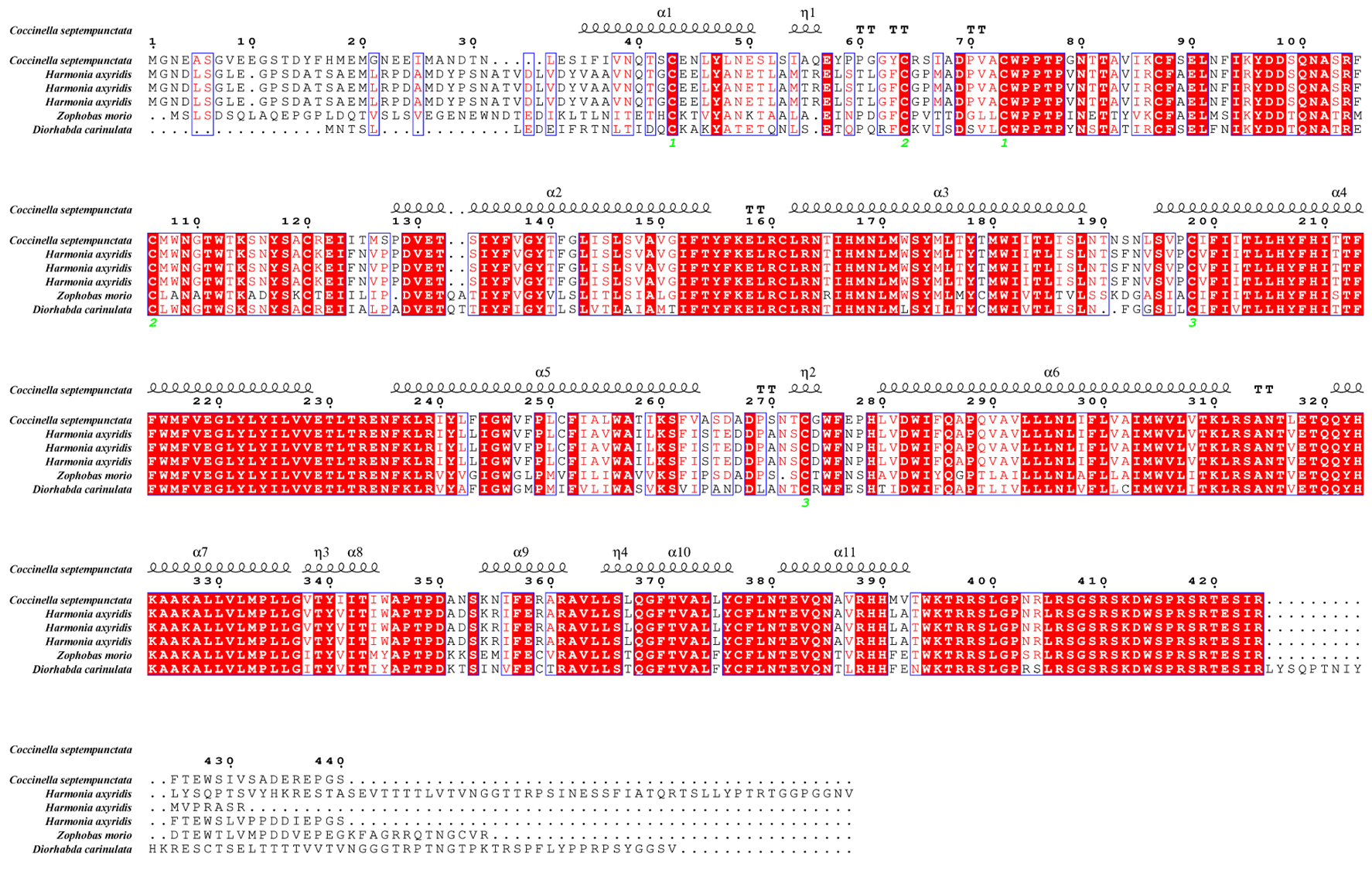


**Figure S6：** **The amino acid sequence alignment of** **DH receptor in *C. septempunctata***


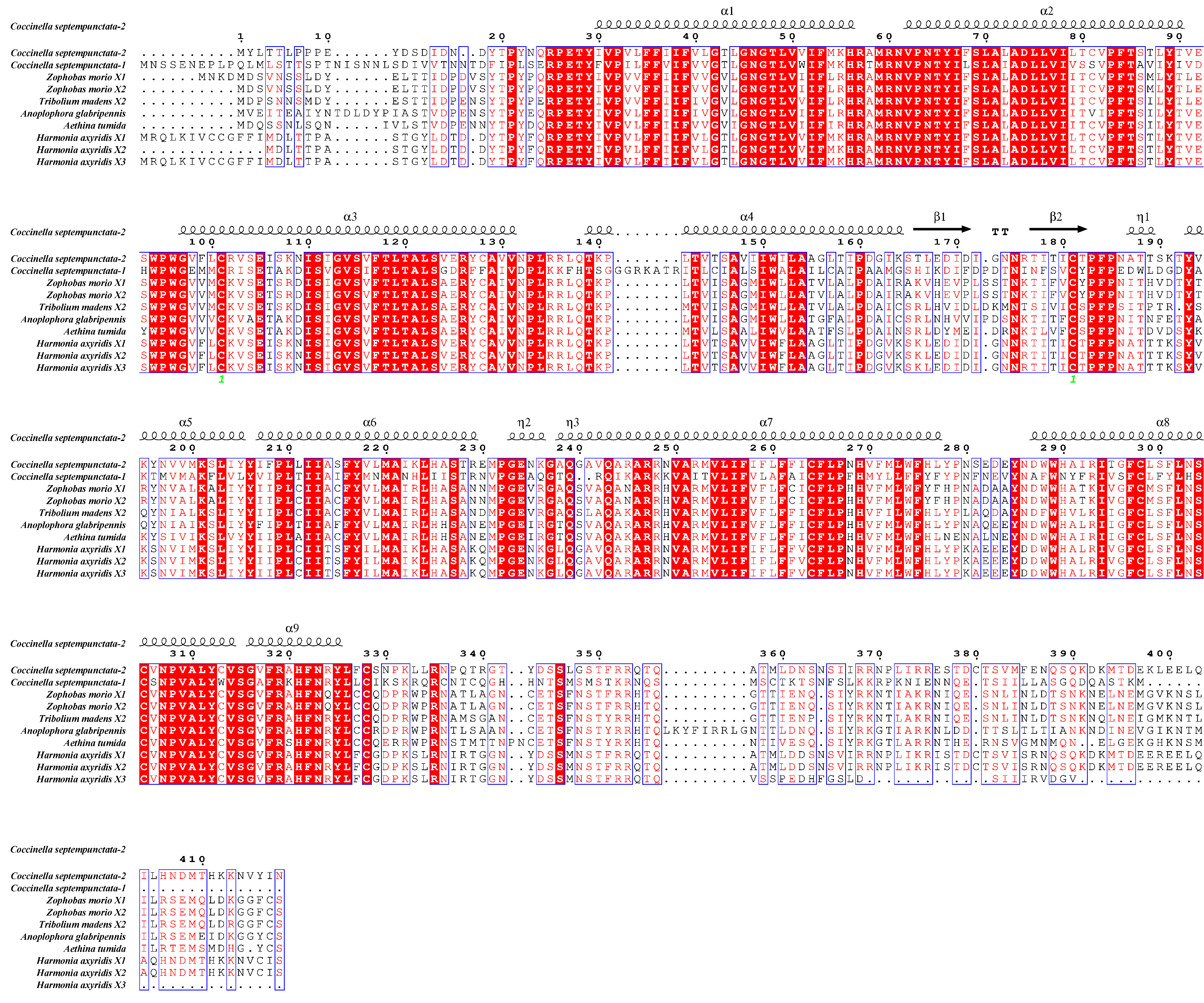


**Figure S7：** **The amino acid sequence alignment of** **CCHamide receptor in *C. septempunctata***


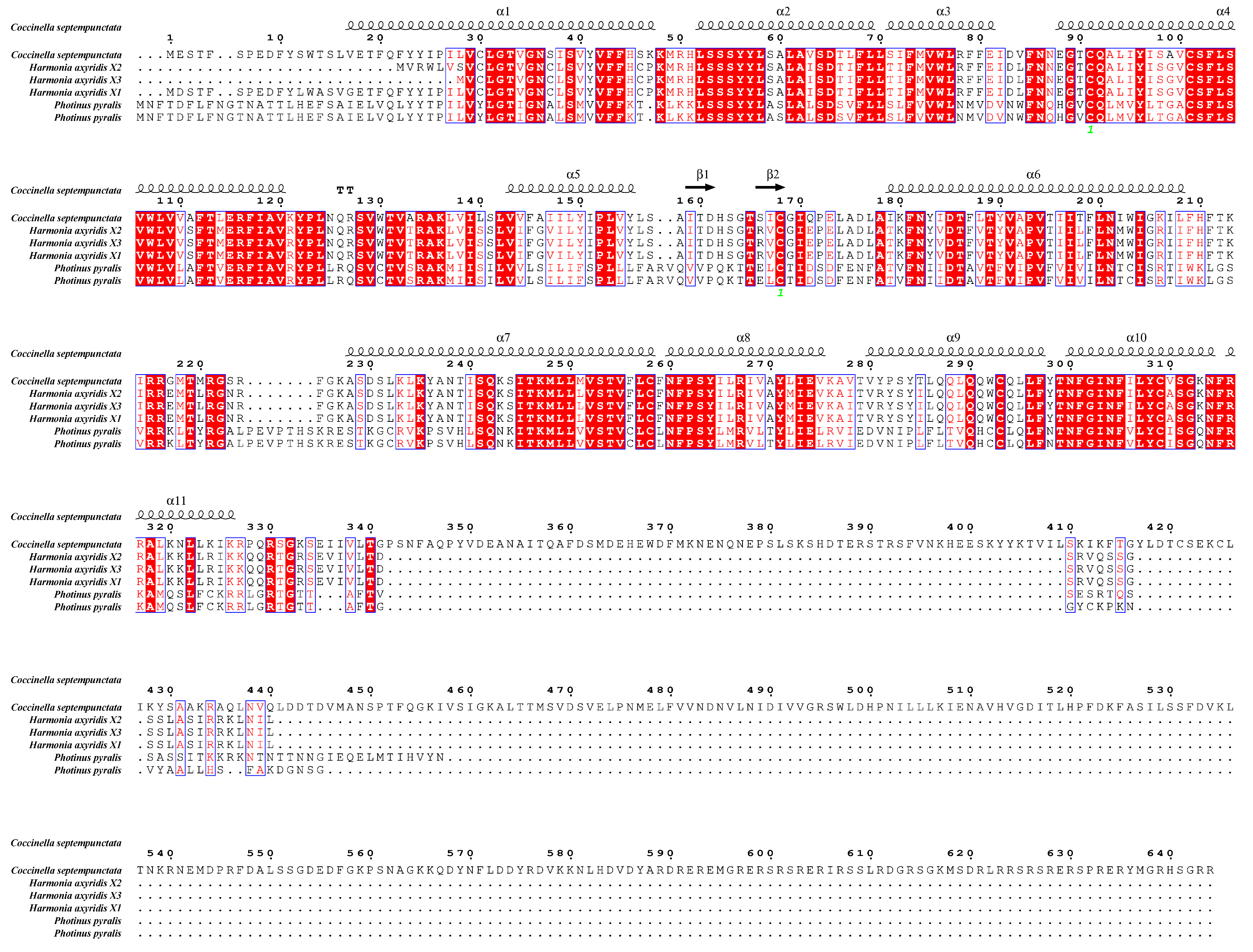


**Figure S8：** **The amino acid sequence alignment of** **CAPA receptor in *C. septempunctata***


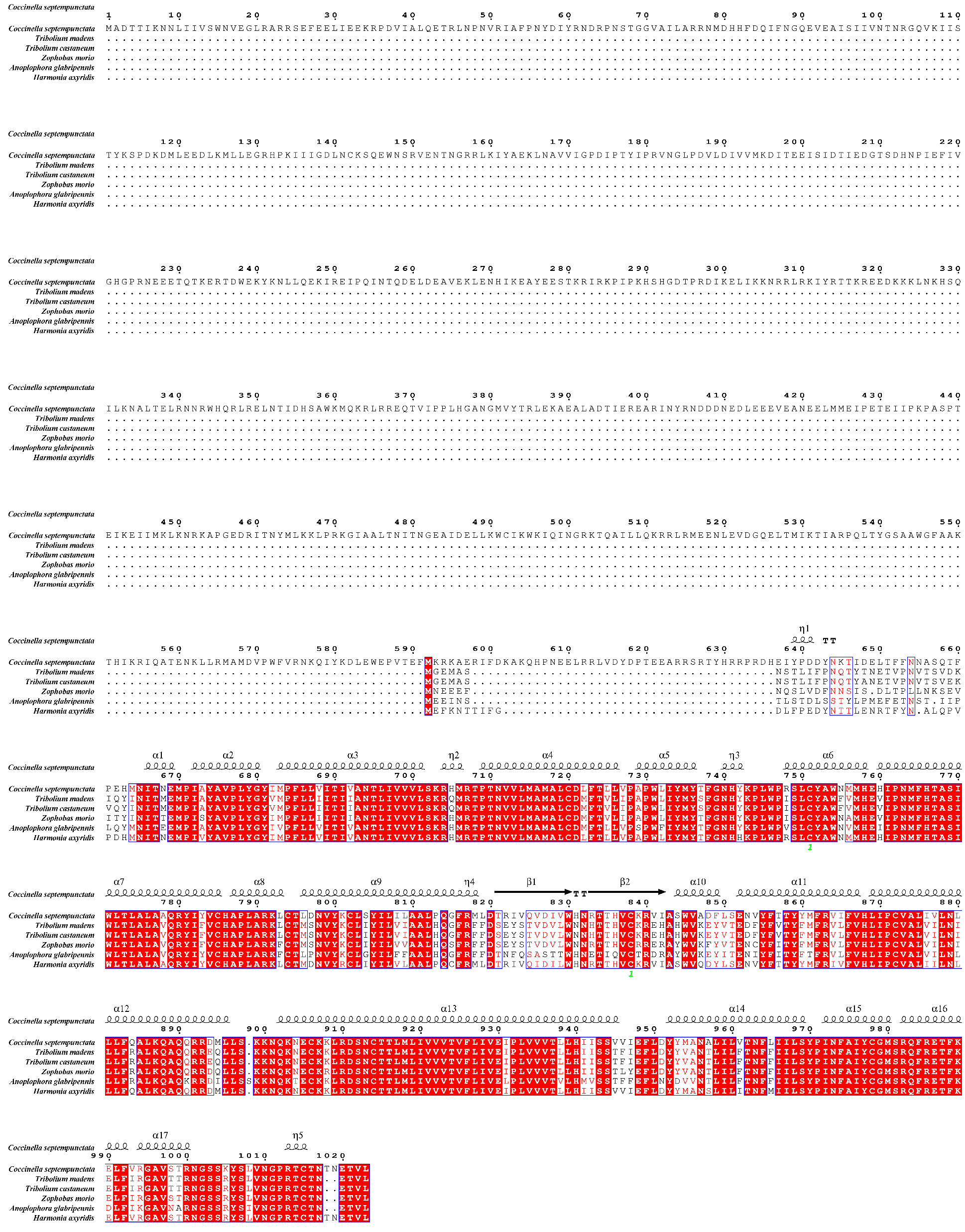


**Figure S9：** **The amino acid sequence alignment of SP receptor in** ***C. septempunctata***
